# Sexual orientation is associated with 2D:4D finger length ratios in both sexes: an updated and expanded meta-analysis

**DOI:** 10.3389/fpsyg.2025.1559158

**Published:** 2025-04-25

**Authors:** Ashlyn Swift-Gallant, Toe Aung, Stephanie Salia, S. Marc Breedlove, David Puts

**Affiliations:** ^1^Department of Psychology, Memorial University of Newfoundland and Labrador, St. John’s, NL, Canada; ^2^Department of Psychology and Counseling, Immaculata University, Immaculata, PA, United States; ^3^Neuroscience Program, Michigan State University, East Lansing, MI, United States; ^4^Departments of Anthropology and Psychology, Pennsylvania State University, University Park, PA, United States

**Keywords:** sexual orientation, bisexuality, digit ratios, 2D:4D, prenatal androgens, prenatal estrogens

## Abstract

The ratio of the lengths of the 2nd and 4th fingers (2D:4D) is a putative marker for prenatal gonadal hormone signaling and has been linked to human sexual orientation. Although 2D:4D is consistently found to be lower in males than females, the association with sexual orientation is variable across studies, with one meta-analysis finding lower (more masculine) digit ratios in lesbians than heterosexual females, but no overall association in males. However, this previous meta-analysis considered neither unpublished datasets nor bisexual individuals separately from homosexual and heterosexual individuals. Moreover, 17 datasets examining relationships between 2D:4D and sexual orientation have been published since that time, and we located an additional 11 unpublished datasets. We therefore conducted an updated and expanded meta-analysis comprising 51 studies, including 44 male and 34 female datasets, totaling 227,648 participants. This meta-analysis also explored whether 2D:4D differed between heterosexual and bisexual and/or non-exclusive individuals in both sexes. Results indicate lower (more male-typical) digit ratios in homosexual women (right hand *g =* 0.26, left hand *g =* 0.16; both adjusted following trim-and-fill), and higher (more female-typical) ratios in homosexual men (right hand *g =* −0.17, left hand *g =* −0.20; both adjusted) compared to heterosexual same-sex counterparts. Moderator analyses do not support publication bias for females. For males, positive findings were more likely to be published, but robustness tests, including trim-and-fill and leave-one-out, support the findings’ robustness. No significant differences were observed in 2D:4D between male or female bisexual and heterosexual individuals. These findings are consistent with evidence that prenatal androgens increase attraction to females and/or that prenatal estrogens increase attraction to males.

## Introduction

Perinatal androgens play a central role in shaping sex differences in the brain and behavior across mammalian species by regulating patterns of gene expression in the developing brain (e.g., Xu et al., 2012). Comparisons of people with and without various endocrine conditions suggest that androgens play a similar role in the development of human sex differences in brain and behavior (Shirazi et al., 2022; Swift-Gallant et al., 2022, 2023). However, disentangling direct effects of androgens on brain development from other biological and/or environmental factors, such as differential treatment by parents or physicians, remains challenging. This problem, coupled with the ethical infeasibility of experimental studies in humans, has led to considerable interest in exploring retrospective biomarkers of early androgen action (Swift-Gallant et al., 2020, 2023).

One such putative biomarker is 2D:4D, the ratio between the lengths of the second (2D) and fourth (4D) manual digits. 2D:4D is consistently lower in males (shorter index finger relative to ring finger; for meta-analysis, see Hönekopp and Watson, 2010), and converging evidence links prenatal androgens to its development (reviewed in Puts et al., 2008; Swift-Gallant et al., 2020, 2023). For example, digit ratios are lower (more male-typical) among women with congenital adrenal hyperplasia, in which prenatal androgens are elevated (Richards et al., 2020a), whereas digit ratios are higher (more female-typical) among chromosomal males with insensitivity to androgens (e.g., androgen insensitivity syndrome; Berenbaum et al., 2009; Van Hemmen et al., 2017).

Some evidence supports a link between 2D:4D and sexual orientation, one of the most strongly sexually differentiated human psychological traits (Balthazart, 2011; Hines, 2011; Kostic and Scofield, 2022). However, reported relationships between 2D:4D and sexual orientation have been mixed, with studies reporting lower ratios, higher ratios, or no significant correlation (reviewed in Swift-Gallant et al., 2020). These varied results prompted a previous meta-analysis (Grimbos et al., 2010), which found lower (i.e., more male-typical) 2D:4D among women with same-sex orientations than their heterosexual counterparts but no association of 2D:4D with male sexual orientation.

**Figure 1:**
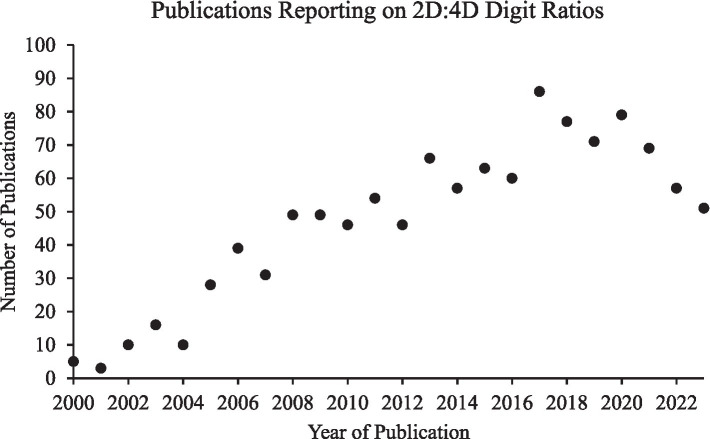
Publications reporting on digit ratios. The number of publications reporting on 2D:4D digit ratios has grown substantially since the last comprehensive meta-analysis assessing this measure in relation to sexual orientation in 2010 (Grimbos et al., 2010). A search on PubMed using the keywords “2D:4D” or “2D4D” or “digit ratio” for years 2000–2023 yielded the above number of publications by year (search date: November 26, 2024).

Despite its contribution, the Grimbos et al. (2010) meta-analysis did not include unpublished datasets, leaving it vulnerable to the “file drawer problem” that negative results may be less likely to be published, a concern often cited in critiques of digit ratio research (e.g., McCormick and Carré, 2020). Grimbos et al. also treated sexual orientation dichotomously, collapsing bisexual individuals with those exclusively oriented toward same-sex partners, and thus could not test whether bisexual individuals are intermediate between heterosexual and homosexual orientations or more similar to either. Additionally, since the last meta-analysis by Grimbos et al. (2010), there has been a significant increase in 2D:4D research, with the number of publications more than doubling between 2010 and 2020 (Figure 1). We have identified 44 datasets (10 unpublished) for male sexual orientation and 34 datasets (5 unpublished) for female sexual orientation, an increase of 26 male and 21 female datasets from those included in Grimbos et al. (2010). We therefore conducted a new meta-analysis on this larger sample that includes unpublished data to mitigate the effects of publication bias, consider intermediate sexual orientations, increase the precision of effect size estimates, and better assess the robustness of any associations.

## Methods

Following Preferred Reporting Items for Systematic Reviews and Meta-Analyses (PRISMA) guidelines (Page et al., 2021), we conducted a systematic literature search and extracted pertinent data from 60 published studies using two prominent electronic databases, PubMed and Google Scholar. In addition to consulting published works, we reached out to researchers who have published on 2D:4D, regardless of whether they evaluated this marker in relation to sexual orientation. Using keywords such as “2D:4D” and “digit ratio,” we identified 296 unique corresponding authors with publications using this marker. From these authors, we requested data on sexual orientation for their published datasets where this information was originally omitted. Simultaneously, we inquired about any unpublished datasets containing the requisite information.

**Figure 2:**
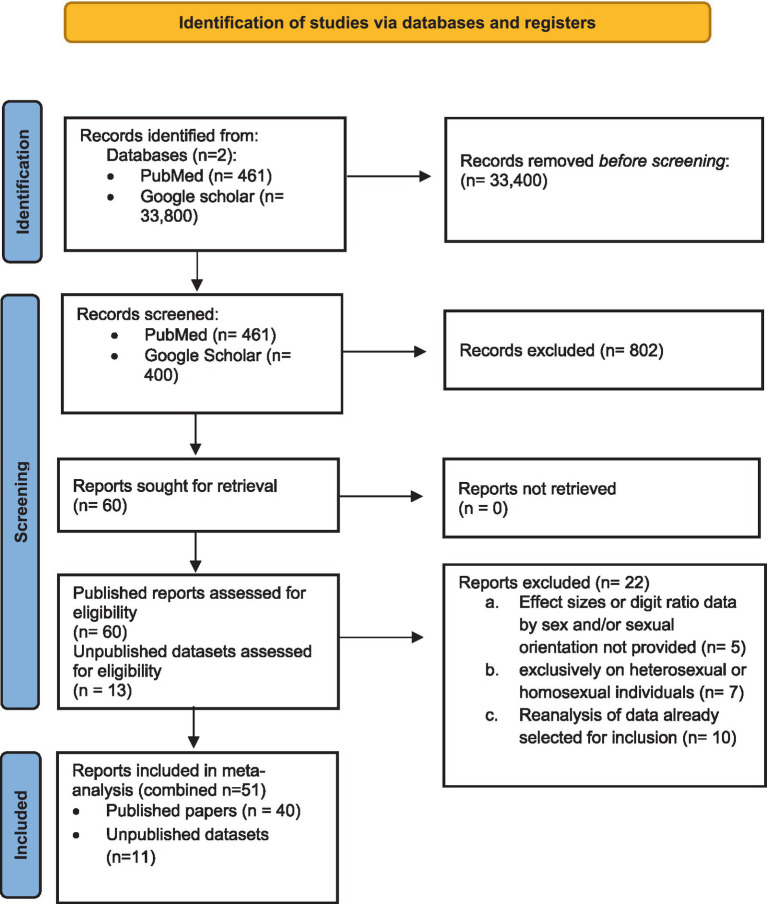
Prisma Flow Chart summarizing the records retrieval and workflow.

Articles and unpublished data were eligible for inclusion if they reported data on 2D:4D by sex and sexual orientation. Studies were excluded if they did not report an effect size or mean 2D:4D and standard deviation (SD) or standard error (SE), broken down by sex and sexual orientation. Studies were also excluded if they focused exclusively on heterosexual or homosexual individuals, or used or reanalyzed previously published data that had already been selected for inclusion. Out of 60 published papers and 13 unpublished datasets assessed for eligibility, 40 published studies and 11 unpublished datasets (comprising 10 male and 5 female unpublished data sets) were determined to be suitable for inclusion in the meta-analysis (i.e., 22 were excluded; see Supplementary Table S1), comprising information from a total of 227,648 participants (Figure 2). In addition to recording effect sizes for right and left hand 2D:4D from these studies, we recorded methodologically relevant variables and study characteristics for planned moderation analysis, including publication status (published or unpublished) geographic location (North American, UK/Europe, Asia, or other) and digit measurement method (direct, self-report, photocopy/scan, mixed, or unknown). The collated data including, effect sizes and moderator variables, are provided as a supplementary file.

### Search strategy and study selection

We conducted an article search from 2000 to 2024 using PubMed and Google Scholar. Two search strategies were employed on PubMed using the terms: (1) (((((2D:4D and Sexual Orientation)); and (2) ((((((Digit ratio) OR (2D:4D)) OR (2D4D)) OR (finger length)) OR (digit length)) OR (finger) OR (digit)) AND ((((sexual orientation) OR (lesbian)) OR (bisexual)) OR (heterosexual)). Similarly, on Google Scholar, the search included the terms: (1) “2D:4D and Sexual Orientation” and (2) “Digit ratio”|“2D:4D”|“2D4D”|“finger length”|“digit length”|“finger”|“digit” and “lesbian”|“bisexual”|“sexual orientation”|“heterosexual.” PubMed retrieved a combined total of 461 reports, while Google Scholar yielded 33,800 reports; all Pubmed studies and the top 400 articles returned by Google Scholar were reviewed for inclusion. Additionally, we contacted the corresponding authors (*n* = 296) of 2D:4D studies to request information on the sexual orientation of participants, as well as inquire about unpublished data on both 2D:4D and sexual orientation of participants.

### Statistical analyses

Meta-analyses were conducted using a random effects model implemented via the “metafor” package (version 4.2–0; Viechtbauer, 2010) in *R* (version 4.3.0). Standardized effect sizes were calculated using Hedges’s *g* via the “escalc” function, and the random effects models were tested with the “rma.mv” function. Additionally, leave-one-out analyses were conducted utilizing the “leave1out” function to investigate the robustness of results and their dependence on any individual study. To address publication bias, Duval and Tweedie’s trim-and-fill tests were applied using the “trimfill” function. The data and analysis scripts for all tested models are accessible in the supplementary file.

Following Grimbos et al. (2010), we excluded Manning et al. (2007) from primary analyses due to its potential to exert undue influence on meta-analytic results because of its size (>200,000 participants). Results of analyses including Manning et al. (2007) are reported in section Supplementary Results.

## Results

### Sex differences in digit ratios

**Figure 3:**
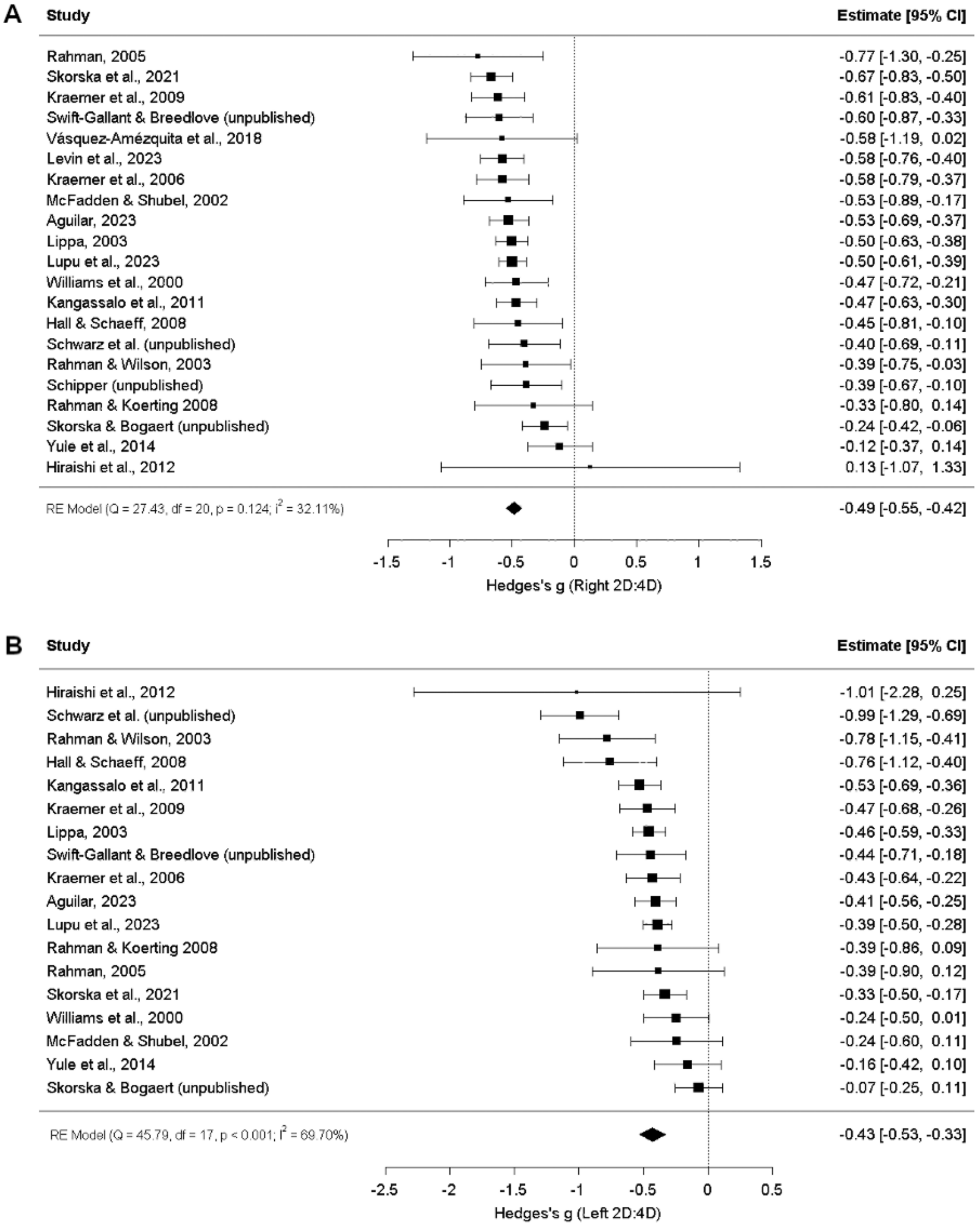
Right **(A)** and Left **(B)** 2D:4D comparison between exclusively heterosexual men and exclusively heterosexual women (Rahman, 2005; Skorska et al., 2021; Kraemer et al., 2009; Vásquez-Amézquita et al., 2018; Levin et al., 2023; Kraemer et al., 2006; McFadden and Shubel, 2002; Aguilar, 2023; Lippa, 2003; Lupu et al., 2023; Williams et al., 2000; Kangassalo et al., 2011; Hall and Schaeff, 2008; Rahman and Wilson, 2003; Rahman and Koerting, 2008; Yule et al., 2014; Hiraishi et al., 2012).

Digit ratios exhibited expected sex differences: Heterosexual men had lower 2D:4D than heterosexual women for both the right hand (*g =* −0.49, *p* < 0.001; Figure 3) and left hand (*g =* −0.43, *p* < 0.001).

### Male sexual orientation and digit ratios

#### Exclusive heterosexual vs. exclusive homosexual men

##### Right 2D:4D

**Figure 4:**
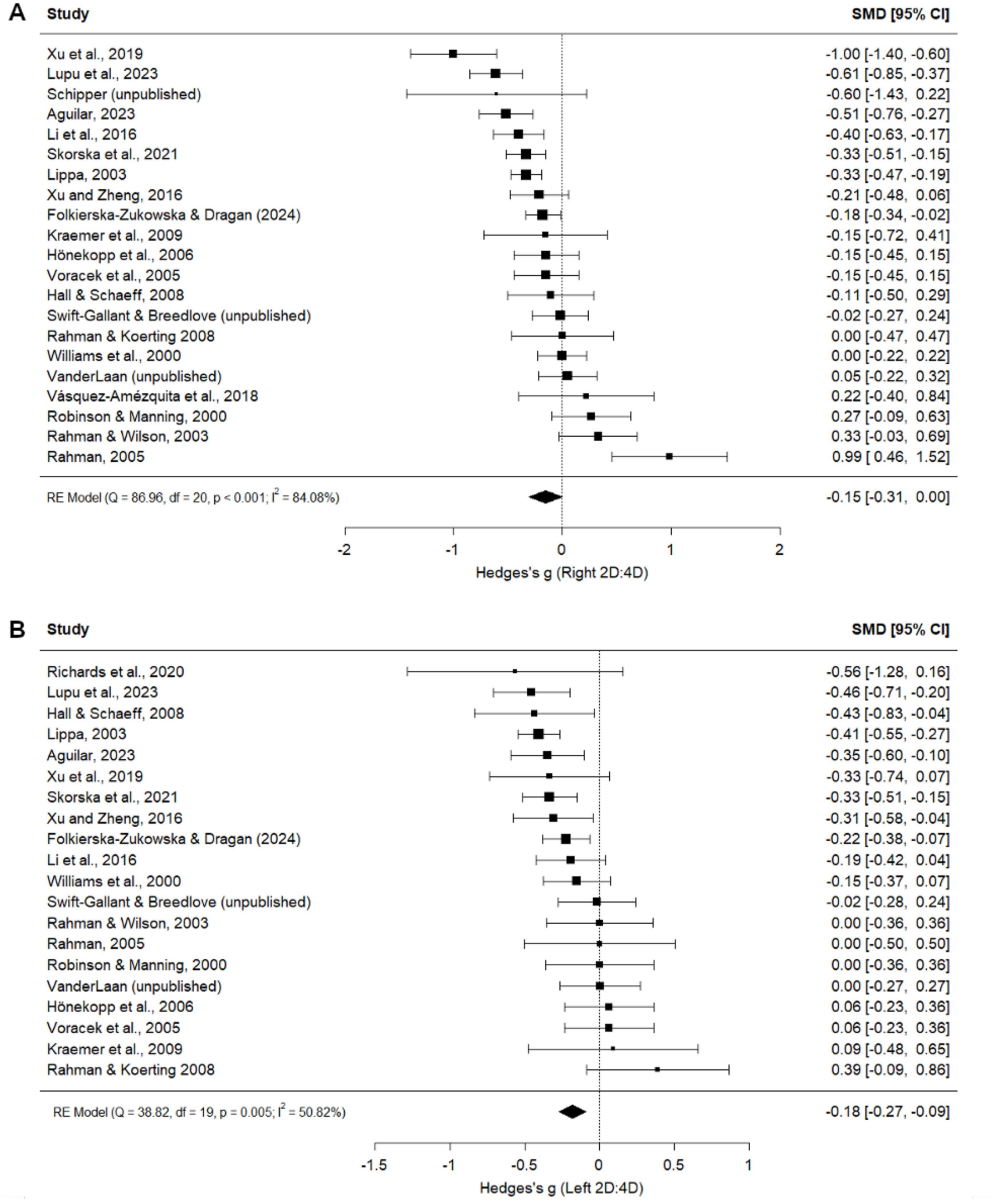
Right **(A)** and Left **(B)** 2D:4D comparison between exclusively heterosexual men and exclusively homosexual men (Xu et al., 2019; Lupu et al., 2023; Aguilar, 2023; Li et al., 2016; Skorska et al., 2021; Lippa, 2003; Xu and Zheng, 2016; Folkierska-Żukowska and Dragan, 2024; Kraemer et al., 2009; Hönekopp et al., 2006; Voracek et al., 2005; Hall and Schaeff, 2008; Rahman and Koerting, 2008; Williams et al., 2000; Vásquez-Amézquita et al., 2018; Robinson and Manning, 2000; Rahman and Wilson, 2003; Rahman, 2005; Richards et al., 2020b).

Exclusively heterosexual and homosexual men did not significantly differ in right hand 2D:4D (*g =* −0.15, *p* = 0.051; Figure 4A). Leave-one-out analysis produced Hedge’s *g* values ranging from −0.12 to −0.20 (Supplementary Figures S1–S4). No significant moderators were found for the right hand heterosexual and homosexual comparisons (Table 1). However, trim-and-fill analysis imputed seven studies and excluded one (Rahman, 2005), leading to an adjusted point estimate where 2D:4D is lower in heterosexual men than in homosexual men (*g* = −0.17, *p* < 0.001; Supplementary Figures S5–S10).

**Table 1:** Results from moderator analyses for exclusive heterosexual men vs. exclusive homosexual men.

Right 2D:4D model	*Q*_m_	*k*	*g*	se	*z*	*p*	Lower CI	Upper CI
Geographic location	4.22					0.238		
North America		8	−0.10	0.13	−0.78	0.434	−0.37	0.16
UK/Europe		10	−0.13	0.12	−1.09	0.275	−0.37	0.11
Other^+^								
Asia		3	−0.32	0.20	−1.55	0.120	−0.72	0.08
Measurement type	6.78					0.079		
Direct		1	−0.02	0.13	−0.16	0.871	−0.28	0.24
Self-report^+^								
Photocopy/scan		12	−0.27	0.10	−2.59	0.010	−0.47	−0.07
Mixed or unknown		2	0.05	0.25	0.20	0.838	−0.44	0.54
Publication status	3.66					0.161		
Published		18	−0.16	0.09	−1.87	0.062	−0.33	0.01
Unpublished		3	−0.09	0.23	−0.42	0.674	−0.54	0.35

Left 2D:4D model	*Q*_m_	*k*	*g*	se	*z*	*p*	Lower CI	Upper CI
Geographic location^1^	17.59					0.001		
North America		7	−0.22	0.07	−2.96	0.003	−0.37	−0.07
UK/Europe		10	−0.10	0.07	−1.34	0.179	−0.24	0.05
Other^+^								
Asia		3	−0.28	0.11	−2.65	0.008	−0.49	−0.07
Measurement type^1^	20.02					0.001		
Direct		7	−0.19	0.08	−2.46	0.014	−0.33	−0.04
Self-report		1	−0.56	0.39	−1.44	0.150	−1.33	0.20
Photocopy/scan		10	−0.21	0.06	−3.44	0.001	−0.33	−0.09
Mixed or unknown		2	0.04	0.15	0.24	0.811	−0.26	0.33
Publication status^1^	21.28					<0.001		
Published		18	−0.21	0.05	−4.61	<0.0001	−0.30	−0.12
Unpublished		2	−0.01	0.13	−0.06	0.949	−0.26	0.24

Pairwise comparisons were tested for the model with a significant moderator. ^1^No significant difference was observed for all pairwise comparisons (*p* > 0.05). ^+^Not identified in data.

##### Left 2D:4D

Exclusively heterosexual men had a lower left hand 2D:4D than exclusively homosexual men (*g =* −0.18, *p* < 0.001; Figure 4B). Leave-one-out analyses produced Hedge’s *g* values ranging from −0.16 to −0.20, suggesting that the difference between exclusive heterosexual and homosexual men does not depend on the inclusion of any particular study (Supplementary Figure S2). This relationship was moderated by publication status and present only in published studies, suggesting a tendency for statistically significant effects to be published (Table 1). Two missing studies were imputed during trim-and-fill analysis (Supplementary Figure S8), leading to a point estimate of *g =* −0.20. Geographical location, measurement type, and publication status were significant moderators of left hand digit ratios, but no pairwise comparisons were significant (Table 1).

#### Heterosexual vs. non-heterosexual men

##### Right 2D:4D

**Figure 5:**
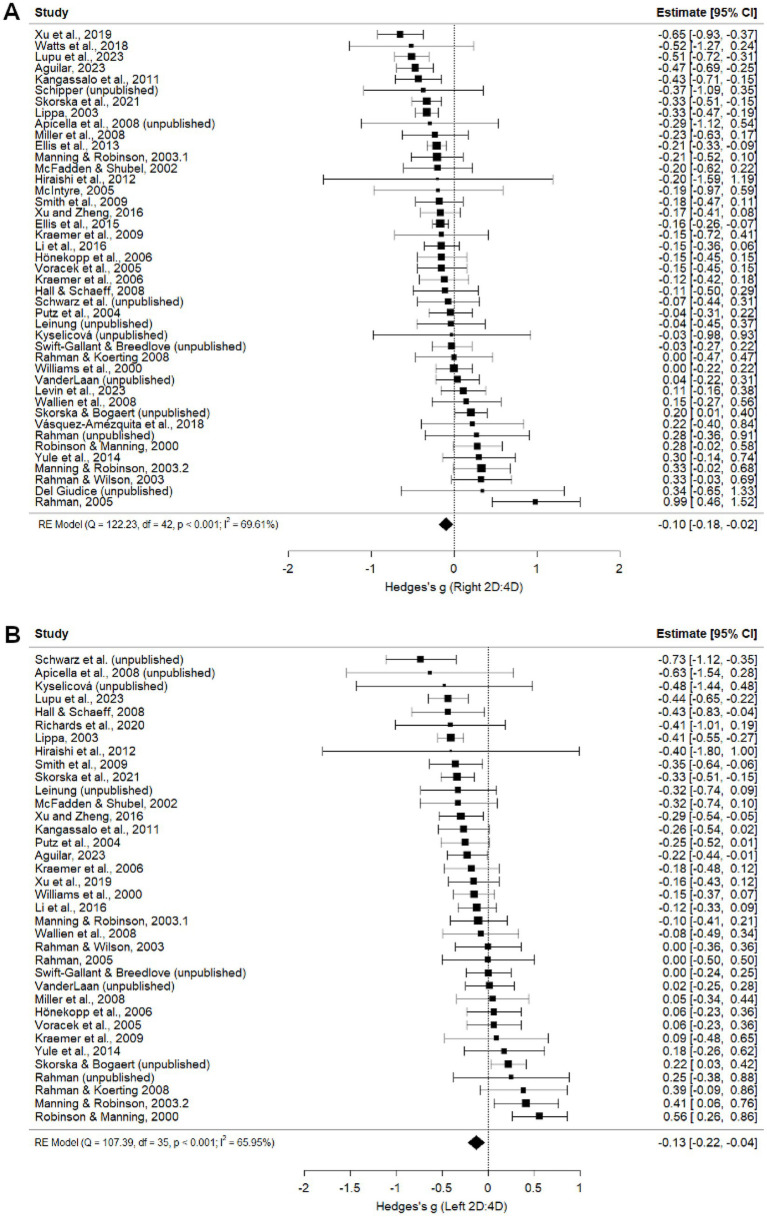
Right **(A)** and Left **(B)** 2D:4D comparison between heterosexual men and non-heterosexual men. 1 = UK participants; 2 = Multi-ethnic participants. In the random-effects model where Manning and Robinson (2003) studies were treated as two samples rather than one study, right 2D:4D ratios (*g =* −0.10 [−0.18, −0.01], *p* = 0.025) differed significantly between heterosexual men and non-heterosexual men, while left 2D:4D ratios (*g =* −0.12 [−0.21, −0.03], *p* = 0.010) do not differ significantly between heterosexual men and non-heterosexual men (Xu et al., 2019; Watts et al., 2018; Lupu et al., 2023; Aguilar, 2023; Kangassalo et al., 2011; Skorska et al., 2021; Lippa, 2003; Miller et al., 2008; Ellis et al., 2013; Manning and Robinson, 2003; McFadden and Shubel, 2002, Hiraishi et al., 2012; McIntyre, 2005; Smith et al., 2010; Xu and Zheng, 2016; Ellis et al., 2015; Kraemer et al., 2009; Li et al., 2016; Hönekopp et al., 2006; Voracek et al., 2005; Kraemer et al., 2006; Hall and Schaeff, 2008; Putz et al., 2004; Rahman and Koerting, 2008; Williams et al., 2000; Levin et al., 2023; Wallien et al., 2008; Vásquez-Amézquita et al., 2018; Robinson and Manning, 2000; Yule et al., 2014; Rahman and Wilson, 2003; Rahman, 2005; Richards et al., 2020b).

Exclusively heterosexual men had a lower right 2D:4D than non-heterosexual (bisexual plus homosexual) men (*g* = −0.10, *p* = 0.018; Figure 5A). This relationship was present in published studies, whereas the point estimate in unpublished studies was near zero, suggesting a tendency for statistically significant effects to be published (Table 2). However, following trim-and-fill analyses, which imputed seven studies and excluded one (Rahman, 2005), the relationship remained significant, and the effect size increased (adjusted *g* = −0.17, *p* < 0.001; Supplementary Figure S9). Leave-one-out analyses produced Hedge’s g values ranging from −0.08 to −0.11, suggesting that difference between heterosexual and non-heterosexual men is robust to the exclusion of individual studies (Supplementary Figure S3). Measurement type was a significant moderator, with a significant difference between photocopy/scan and mixed or unknown measures, suggesting that mixed methods or studies that did not report how they measured ratios were more likely to find higher right 2D:4D among heterosexual men than non-heterosexual men (Table 2).

**Table 2:** Results from moderator analyses for heterosexual men vs. non-heterosexual men.

Right 2D:4D model	*Q*_m_	*k*	*g*	se	*z*	*p*	Lower CI	Upper CI
Geographic location	6.49					0.166		
North America		18	−0.06	0.06	−0.99	0.320	−0.19	0.06
UK/Europe		19	−0.10	0.07	−1.54	0.124	−0.24	0.03
Other		2	−0.18	0.26	−0.71	0.477	−0.69	0.32
Asia		4	−0.22	0.14	−1.62	0.105	−0.49	0.05
Measurement type^1^	13.05					0.011		
Direct		6	0.06	0.10	0.55	0.580	−0.15	0.26
Self-report		4	−0.11	0.11	−0.99	0.320	−0.33	0.11
Photocopy/scan		28	−0.17	0.05	−3.25	0.001	−0.27	−0.07
Mixed or unknown		4	0.17	0.15	1.10	0.273	−0.13	0.47
Publication status^2^	8.40					0.015		
Published		33	−0.13	0.05	−2.89	0.004	−0.22	−0.04
Unpublished		10	0.02	0.10	0.25	0.800	−0.16	0.21

Left 2D:4D model		*k*	*g*	se	*z*	*p*	Lower CI	Upper CI
Geographic location^2^	9.79					0.044		
North America		13	−0.18	0.08	−2.36	0.019	−0.33	−0.03
UK/Europe		7	−0.06	0.07	−0.81	0.416	−0.19	0.08
Other		2	−0.07	0.26	−0.28	0.780	−0.58	0.43
Asia		4	−0.25	0.14	−1.87	0.062	−0.52	0.01
Measurement type^3^	22.38					<0.001		
Direct		6	−0.04	0.09	−0.43	0.667	−0.23	0.14
Self-report		2	−0.13	0.17	−0.75	0.454	−0.45	0.20
Photocopy/scan		23	−0.21	0.05	−4.09	<0.001	−0.30	−0.11
Mixed or unknown		4	0.31	0.14	2.21	0.027	0.04	0.59
Publication status^2^	7.43					0.024		
Published		28	−0.13	0.05	−2.49	0.013	−0.23	−0.03
Unpublished		8	−0.12	0.11	−1.11	0.267	−0.33	0.09

Pairwise comparisons were tested for the model with a significant moderator. ^1^Significant difference was observed between photocopy/scan and mixed or unknown measures (*p* = 0.038). ^2^No significant difference was observed for all pairwise other comparisons (*p* > 0.05). ^3^Significant difference was observed between direct and mixed or unknown measures (*p* = 0.038), between self-reported measures and mixed or unknown measures (*p* = 0.046), and between photocopy/scan and mixed or unknown measures (*p* < 0.001).

##### Left 2D:4D

Exclusively heterosexual men also had a lower left hand 2D:4D than non-heterosexual (bisexual plus homosexual) men (*g* = −0.13, *p* = 0.006; Figure 5B). Publication status was a significant moderator; however, the point estimate was nearly identical for published (*g =* −0.13, *p* = 0.013) and unpublished (*g =* −0.12, *p* = 0.267) studies, and no studies were imputed in trim-and-fill analysis. Leave-one-out analyses for the left hand produced Hedge’s *g* values ranging from −0.11 to −0.15, suggesting these findings are robust to the exclusion of any particular study (Supplementary Figure S4). Measurement type moderated left hand comparisons, with significant differences observed between direct and mixed or unknown measures, between self-reported measures and mixed or unknown measures, and between photocopy/scan and mixed or unknown measures (Table 2). The effect size was in the opposite direction for studies reporting mixed or unknown measurement methods compared to direct, self-report and photocopy/scan methods. Geographical location moderated relationships, but no pairwise comparisons reached significance.

#### Comparisons of heterosexual, bisexual, and homosexual men

We also tested whether relationships between sexual orientation and 2D:4D differed across comparisons between heterosexual and bisexual men, bisexual and homosexual men, and heterosexual and homosexual men across the 8 samples for which these comparisons were possible (Supplementary Table S4). In left 2D:4D, homosexual men had a higher (more female-typical) 2D:4D than heterosexual men, whereas bisexual men differed from neither heterosexual nor homosexual men. A similar non-significant trend was evident for right 2D:4D.

### Female sexual orientation and digit ratios

#### Exclusive heterosexual vs. exclusive homosexual women

##### Right 2D:4D

**Figure 6:**
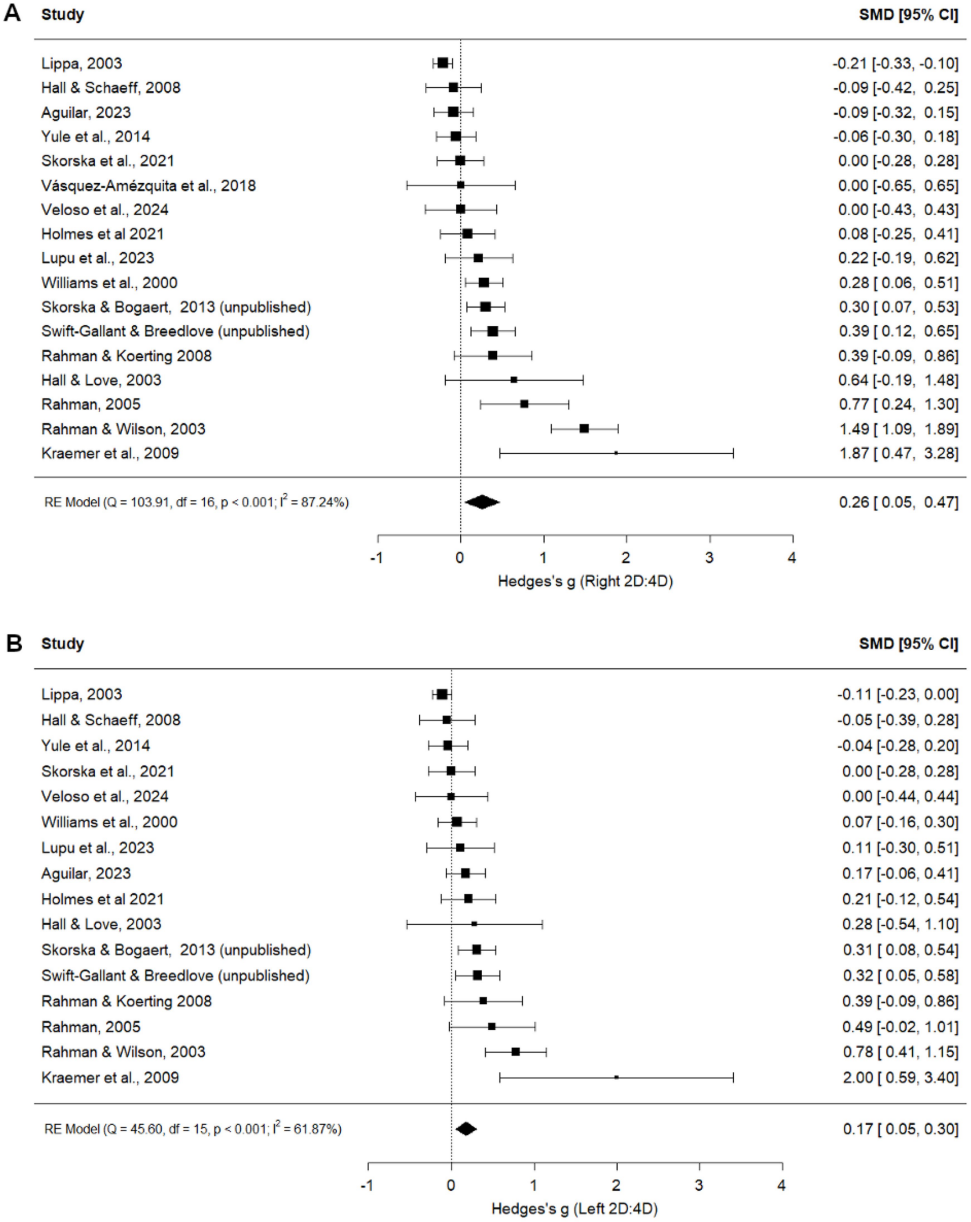
Right **(A)** and Left **(B)** 2D:4D comparison between exclusively heterosexual women and exclusively homosexual women (Lippa, 2003; Hall and Schaeff, 2008; Aguilar, 2023; Yule et al., 2014; Skorska et al., 2021; Vásquez-Amézquita et al., 2018; Veloso et al., 2024; Holmes et al., 2021; Lupu et al., 2023; Williams et al., 2000; Rahman and Koerting, 2008; Hall and Love, 2003; Rahman, 2005; Rahman and Wilson, 2003; Kraemer et al., 2009).

Exclusively heterosexual women had a higher right 2D:4D than exclusively homosexual women (*g =* 0.26, *p* = 0.016; Figure 6A). No moderators, including publication status, were significant (Table 3), and no studies were imputed or removed in the trim-and fill analysis (Supplementary Figure S8). Leave-one-out analysis produced Hedge’s *g* values ranging from 0.14 to 0.30 (Supplementary Figures S13).

**Table 3:** Results from moderator analyses for exclusive heterosexual women vs. exclusive homosexual women.

Right 2D:4D model	*Q*_m_	*k*	*g*	se	*z*	*p*	Lower CI	Upper CI
Geographic location	9.21					0.056		
North America		8	0.13	0.16	0.84	0.401	−0.17	0.44
UK/Europe		7	0.52	0.18	2.92	0.004	0.17	0.87
Other		1	0.00	0.45	0.00	1.000	−0.89	0.89
Asia		1	0.00	0.42	0.00	1.000	−0.83	0.83
Measurement type	6.20					0.111		
Direct		7	0.25	0.18	1.39	0.163	−0.10	0.60
Self-report		1	−0.06	0.44	−0.13	0.898	−0.91	0.80
Photocopy/scan		9	0.32	0.16	2.01	0.044	0.01	0.63
Mixed or unknown^+^								
Publication status	5.65					0.059		
Published		15	0.25	0.12	2.08	0.037	0.01	0.48
Unpublished		2	0.34	0.30	1.15	0.252	−0.24	0.93
								

**Left 2D:4D model**	***Q***_**m**_	***k***	***g***	**se**	***z***	***p***	**Lower CI**	**Upper CI**
Geographic location^2^	13.67					0.008		
North America		7	0.08	0.08	0.99	0.320	−0.08	0.24
UK/Europe		7	0.37	0.10	3.56	<0.001	0.17	0.57
Other		1	0.00	0.28	0.00	1.000	−0.55	0.55
Asia		1	0.00	0.22	0.00	1.000	−0.43	0.43
Measurement type^1^	8.01					0.046		
Direct		7	0.17	0.11	1.59	0.111	−0.04	0.39
Self-report		1	−0.04	0.24	−0.17	0.867	−0.51	0.43
Photocopy/scan		8	0.22	0.09	2.34	0.020	0.03	0.40
Mixed or unknown^+^								
Publication status^1^	8.75					0.013		
Published		14	0.14	0.07	2.13	0.033	0.01	0.28
Unpublished		2	0.31	0.15	2.05	0.040	0.01	0.61

Pairwise comparisons were tested for the model with a significant moderator. ^1^No significant difference was observed for all pairwise other comparisons (*p* > 0.05). ^2^Significant difference was observed between UK/Europe and North America (*p* = 0.031). All other comparisons were non-significant. ^+^Not identified in data.

##### Left 2D:4D

Exclusively heterosexual women also had a higher left 2D:4D than exclusively homosexual women (*g =* 0.17, *p* = 0.006; Figure 6B). Publication status moderated this effect: the effect was larger in unpublished (*g =* 0.31, *p* = 0.040) than published (*g =* 0.14, *p* = 0.033) studies (Table 3), although both effects were significant in the same direction. Following trim-and-fill analyses, no study was imputed and one study was removed (Kraemer et al., 2006), resulting in a significant adjusted main effect (*g =* 0.16, *p* = 0.010; Supplementary Figure S8). Leave-one-out analyses produced Hedge’s *g* values ranging from 0.12 to 0.20, also suggesting that the findings are robust to the exclusion of any particular study (Supplementary Figure S14). Geographical location moderated left-hand digit ratios. Pairwise comparisons identified that UK/Europe differed significantly from North America, with UK/Europe having a larger positive effect size (lower left 2D:4D in exclusive homosexual women compared to heterosexual women) than North America (Table 2). Measurement type was a significant moderator, but no pairwise comparisons were significant.

#### Heterosexual vs. non-heterosexual women

**Figure 7:**
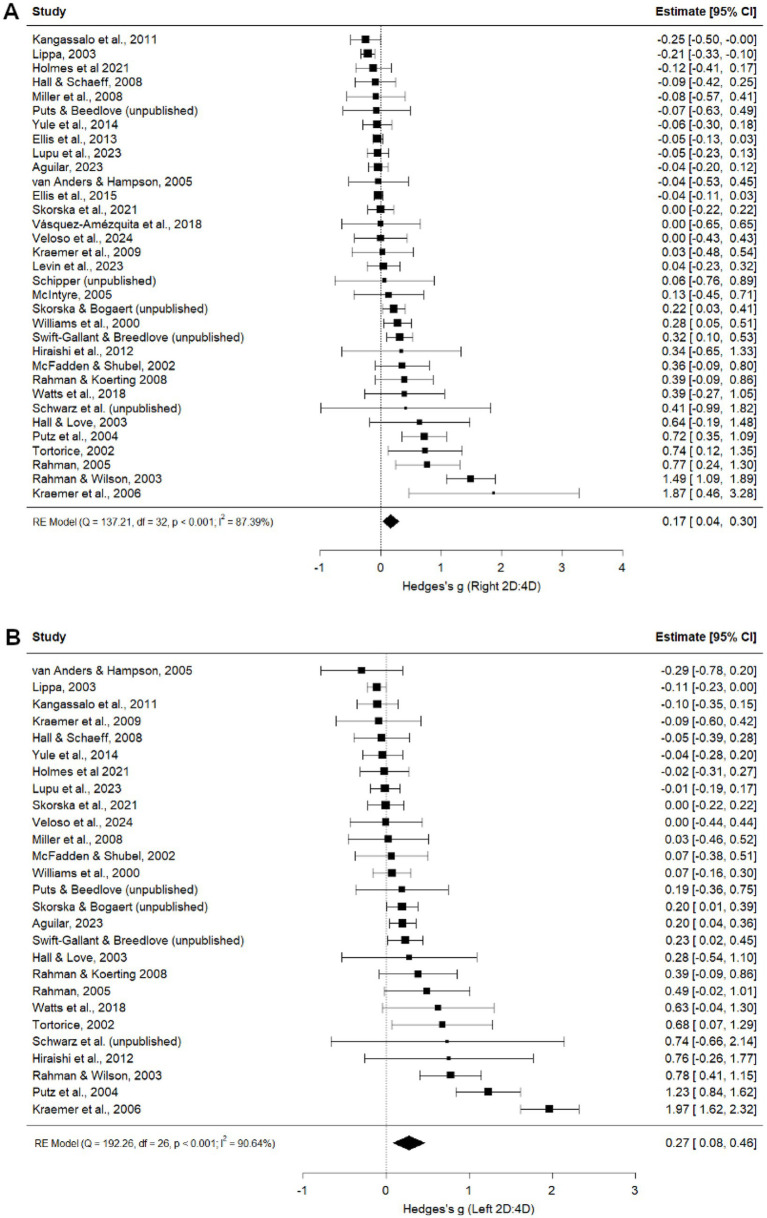
Right **(A)** and Left **(B)** 2D:4D comparison between heterosexual women and non-heterosexual women (Kangassalo et al., 2011; Lippa, 2003; Holmes et al., 2021; Hall and Schaeff, 2008; Miller et al., 2008; Yule et al., 2014; Ellis et al., 2013; Lupu et al., 2023; Aguilar, 2023; Van Anders and Hampson, 2005; Ellis et al., 2015; Skorska et al., 2021; Vásquez-Amézquita et al., 2018; Veloso et al., 2024; Kraemer et al., 2009; Levin et al., 2023; McIntyre, 2005; Williams et al., 2000; Hiraishi et al., 2012; McFadden and Shubel, 2002; Rahman and Koerting, 2008; Watts et al., 2018; Hall and Love, 2003; Putz et al., 2004; Tortorice, 2002; Rahman, 2005; Rahman and Wilson, 2003; Kraemer et al., 2006).

Heterosexual women had a higher digit ratio than non-heterosexual women in both right (*g =* 0.17, *p* = 0.012; Figure 7A) and left (*g =* 0.27, *p* = 0.005; Figure 7B) hands. While publication status was a significant moderator for both hands (Table 4), effect sizes were similar for published and unpublished studies (right hand: published *g =* 0.17, *p* = 0.023; unpublished *g =* 0.18, *p* = 0.324; left hand: published *g =* 0.28, *p* = 0.010; unpublished *g =* 0.26, *p* = 0.333). Following trim-and-fill analyses, no studies were imputed, and one study was removed for the right hand, resulting in an adjusted estimate of *g =* 0.15, *p* = 0.019 (Supplementary Figure S9). For the left hand, six studies were imputed and one study was removed, resulting in a non-significant adjusted estimate *g =* 0.06, *p* = 0.472 (Supplementary Figure S9). Leave-one-out analyses for right (Supplementary Figure S15) and left (Supplementary Figure S16) 2D:4D produced Hedge’s *g* values of 0.09 to 0.18 and 0.18 to 0.29, respectively, indicating that differences between heterosexual and non-heterosexual women are robust to the exclusion of any particular study. Geographical location and measurement type were significant moderators for left, but not right, hand 2D:4D; however, no pairwise comparisons reached significance (Table 4).

**Table 4:** Results from moderator analyses for heterosexual women vs. non-heterosexual women.

Right 2D:4D model	*Q*_m_	*K*	g	se	*z*	*P*	Lower CI	Upper CI
Geographic location	7.82					0.099		
North America		18	0.16	0.09	1.75	0.080	−0.02	0.33
UK/Europe		10	0.27	0.13	2.10	0.036	0.02	0.53
Other		4	−0.03	0.19	−0.14	0.890	−0.41	0.35
Asia		1	0.34	0.60	0.57	0.570	−0.84	1.52
Measurement type^1^	7.71					0.053		
Direct		7	0.22	0.14	1.51	0.131	−0.06	0.50
Self-report		3	−0.05	0.19	−0.26	0.798	−0.42	0.32
Photocopy/scan		23	0.19	0.08	2.31	0.021	0.03	0.36
Mixed or unknown^+^								
Publication status^1^	6.18					0.046		
Published		28	0.17	0.07	2.28	0.023	0.02	0.31
Unpublished		5	0.18	0.19	0.99	0.324	−0.18	0.55

Left 2D:4D model	***Q***_**m**_	***K***	***g***	**se**	***z***	***P***	**Lower CI**	**Upper CI**
Geographic location^1^	11.15					0.025		
North America		12	0.24	0.14	1.70	0.090	−0.04	0.52
UK/Europe		10	0.43	0.16	2.64	0.008	0.11	0.74
Other		4	−0.06	0.25	−0.24	0.809	−0.55	0.43
Asia		1	0.76	0.69	1.10	0.270	−0.59	2.10
Measurement type^1^	8.50					0.037		
Direct		7	0.17	0.20	0.88	0.380	−0.21	0.55
Self-report		1	−0.04	0.48	−0.08	0.934	−0.99	0.91
Photocopy/scan		19	0.33	0.12	2.78	0.006	0.10	0.57
Mixed or unknown^+^								
Publication status^1^	7.62					0.022		
Published		23	0.28	0.11	2.59	0.010	0.07	0.49
Unpublished		4	0.26	0.27	0.97	0.333	−0.27	0.79

Pairwise comparisons were tested for the model with a significant moderator. ^1^No significant difference was observed for all pairwise comparisons (*p* > 0.05). ^+^Not identified in data.

#### Comparisons of heterosexual, bisexual, and homosexual women

We also tested whether relationships between sexual orientation and 2D:4D differed across comparisons between heterosexual and bisexual women, bisexual and homosexual women, and heterosexual and homosexual women across the 6 samples for which these comparisons were possible (Supplementary Table S5). In both hands, heterosexual and bisexual women had higher (more female-typical) 2D:4D than homosexual women but did not differ from each other.

## Discussion

Although relatively few studies were available for comparing heterosexual, bisexual, and homosexual individuals separately, a trend emerged in both sexes: 2D:4D ratios tended to be more similar between bisexual and heterosexual individuals than between either group and homosexual individuals. There was also a tendency for exclusively heterosexual and homosexual individuals to exhibit the greatest differences. These results indicate that the approach used in prior studies comparing heterosexual to non-heterosexual individuals may be less informative than analyses comparing exclusively heterosexual and homosexual individuals. We therefore focus our discussion on comparisons between exclusively heterosexual and homosexual individuals and consider heterosexual/non-heterosexual comparisons in this light.

Our results replicate the main finding from a previous meta-analysis (Grimbos et al., 2010) demonstrating an association between 2D:4D and women’s sexual orientation: Homosexual women tend to have lower (more male-typical) digit ratios in both hands than heterosexual women. However, the inclusion of unpublished data and additional published studies in the present meta-analysis, as well as comparisons of more homogenous groups (exclusive heterosexual vs. homosexual), appears to contribute in two important ways.

First, the present data appear to be less influenced by publication bias than those in Grimbos et al. In the previous meta-analysis, adjusted effect sizes following trim-and-fill (right: 0.13, left: 0.07) were less than half of unadjusted values (right: 0.29, left: 0.23). In the present meta-analysis, the effect size for right 2D:4D (0.26) was unchanged following trim-and-fill, and the difference between adjusted (0.16) and unadjusted (0.17) effects for the left hand was minimal. Second, adjusted effect sizes were approximately twice as large in the present meta-analysis as in Grimbos et al. These results increase confidence that associations between sexual orientation and 2D:4D are real and meaningful (see below). Results from comparisons of heterosexual to non-heterosexual women were also positive but showed greater evidence of publication bias.

In contrast to Grimbos et al. (2010), our findings showed that exclusively homosexual men tend to have higher (more female-typical) 2D:4D ratios than exclusively heterosexual men. This association was statistically significant in the left hand prior to correction for publication bias, and in both hands following trim-and-fill analysis, which also slightly increased effect size estimates from −0.15 to −0.17 (right hand) and from −0.18 to −0.20 (left hand). Somewhat smaller, but statistically significant, relationships were observed in comparisons between heterosexual and non-heterosexual men.

Similar to Grimbos et al. (2010), we excluded Manning et al. (2007) from primary analyses due to its potential to exert undue influence on meta-analytic results because of its size (>200,000 participants). However, this study is included in the Supplementary Results. Notably, the effect sizes remain nearly identical whether or not this study is included. The only exception is the unadjusted right hand comparison of heterosexual and homosexual men: with Manning et al. included, the effect is significant (*p* = 0.045), whereas it is not when excluded (*p* = 0.051); in both cases, the effect size is *g* = −0.15. All other effect sizes differ by *g* = 0.02 or less, with no changes in significance.

It is noteworthy that Manning et al. (2024) conducted a follow-up to their earlier work, Manning et al. (2007). In the original analysis, Manning et al. (2007) compared discrete sexual orientation categories (i.e., homosexual, bisexual, and heterosexual), while the 2024 study assessed sexual attraction scores on a 7-point Likert scale. The 2007 findings revealed significant differences in men, with homosexual and bisexual men exhibiting higher 2D:4D ratios compared to heterosexual men, aligning with the results of the present meta-analyses. However, no significant relationships were identified for women’s sexual orientation categories. Conversely, the 2024 analysis using sexual attraction scores uncovered associations for both men and women, consistent with the findings of the present study. Thus, the association with digit ratio may be obscured when moderately and mostly bisexual individuals are combined. Our present analyses suggest that bisexual women are more similar to heterosexual women in digit ratios, but there may be further nuance, where those falling in the middle of the scale or between heterosexual and bisexual on the scale are more like heterosexual women, while those falling between bisexual and homosexual are more similar to lesbians in digit ratios.

Overall, these results conform to the hypothesis that common endocrine factors influence the development of digit ratios and sexual orientation. Specifically, relatively higher levels of prenatal androgen signaling may simultaneously masculize digit ratios (e.g., Richards et al., 2020a; Swift-Gallant et al., 2020, 2023; Zheng and Cohn, 2011) and increase the probability of gynephilia in females (Puts and Motta-Mena, 2018; Swift-Gallant et al., 2020, 2023). Conversely, relatively lower levels of androgen signaling and/or higher levels of estrogen signaling may feminize digit ratios (e.g., Manning et al., 1998; Zheng and Cohn, 2011) and increase androphilia (Shirazi et al., 2021; Swift-Gallant et al., 2023) in males.

### Addressing concerns of publication bias

To address possible publication bias in the digit ratio literature, we contacted nearly 300 researchers, including those who have published on sexual orientation and those who used digit ratio data in relation to other traits and/or behaviors. We were able to include 10 male and 5 female unpublished datasets in the present meta-analysis. Thus, the primary meta-analyses included both published and unpublished datasets, and to assess potential publication bias we assessed publication status (published vs. unpublished) as a moderator. We also conducted trim-and-fill analyses, and conducted leave-one-out analyses to explore the robustness of results following the exclusion of each individual study.

For female sexual orientation comparisons, our analyses revealed no evidence of publication bias. Publication status was not a significant moderator for the right hand comparisons between heterosexual and homosexual women, and while publication status moderated the left hand comparison, the effect size was larger and in the same direction for unpublished datasets than for published ones (*g =* 0.31 vs. 0.14), which is the opposite of what would be expected if the overall association across published studies were due to publication bias. Similarly, while publication status moderated both the right and left heterosexual and non-heterosexual women comparisons, the effect sizes were nearly identical between published and unpublished studies. Looking to confidence intervals, there was more variability in unpublished datasets, likely due to lower statistical power/smaller sample sizes, which may have contributed to authors’ decisions to not publish. Leave-one-out and trim-and-fill analyses also supported the robustness of these findings.

For male sexual orientation comparisons, publication status moderated many effects, though leave-one-out and trim-and-fill did not render any significant findings non-significant. Indeed, the corrected effect sizes were slightly larger for both right and left hand comparisons between heterosexual versus homosexual men (right *g* = −0.15 vs. adjusted *g =* −0.17; left hand *g* = −0.18 vs. adjusted *g* = −0.20). While unpublished studies tended to have point estimates near zero, suggesting a bias to publish positive results for male sexual orientation measures, additional tests of robustness and publication bias indicated that the combined datasets are an unbiased representation of the studies conducted on this relationship.

### Do sexual orientation effect sizes measure up to sex differences?

Contrary to previous meta-analysis, we found that digit ratios are more female-typical among homosexual and non-heterosexual men compared to heterosexual men, although the effect sizes are small (*g =* −0.10 to −0.17). Effect sizes for female sexual orientation comparisons were slightly larger, ranging from *g =* 0.17 to 0.28 (similar to Grimbos et al., *g =* 0.23–0.29). Because homosexual individuals do not exhibit the pronounced physiological and reproductive differences observed between the sexes (i.e., traits driven by prenatal androgen exposure), effect sizes for sexual orientation comparisons within sexes would be expected to be smaller than the sex difference. Hence, the strengths of associations between digit ratio and sexual orientation within sexes are consistent with the overall medium-sized sex difference in digit ratio (*g =* 0.44–0.5, i.e., see present meta-analysis on heterosexual sex differences and a meta-analysis by Hönekopp and Watson, 2010).

The modest effect sizes among sexual orientation groups may also relate to three non-mutually exclusive factors. First, smaller effect sizes could result from heterogeneity in the biological pathways underlying sexual orientation. Same-sex orientation likely involves multiple factors, including but not limited to prenatal androgen exposure (e.g., Swift-Gallant et al., 2019; VanderLaan et al., 2022). As a result, aggregating individuals with same-sex orientation into a single group may obscure or dilute associations between digit ratios and sexual orientation. Supporting this view, prior research has found digit ratio differences within subgroups of gay men based on receptive and insertive sex roles (Swift-Gallant et al., 2021). Prior work also supports potential subgroups for female sexual orientation, such that more masculine and/or butch-identifying lesbians present with lower (more male-typical) digit ratios than female-typical or femme-identifying lesbians (reviewed in Swift-Gallant et al., 2020, 2023). Thus, effect sizes may be larger for a subgroup of homosexual males and females. Future research may consider subgroups and/or measuring markers of other biological contributors (e.g., genetics, immune activation) in addition to digit ratios to understand the development of human sexual orientation. In any case, future work should consider the effect sizes reported in the present meta-analysis when designing their studies, to ensure they are sufficiently powered.

Second, digit ratios are an imperfect proxy for prenatal androgen exposure (Swift-Gallant et al., 2020). They are likely influenced not only by prenatal androgens but also by prenatal estrogens and genetic and other factors, which must limit their sensitivity to subtle androgen variations (Swift-Gallant et al., 2020, 2023). This limitation is particularly relevant when studying men and raises the possibility of a “ceiling effect,” where once prenatal androgen levels reach the male-typical range sufficient to masculinize digit ratios and/or sexual orientation, additional androgen exposure may not further influence these traits (Swift-Gallant et al., 2023). Consequently, digit ratios and sexual orientation may be more sensitive to variation in prenatal androgens among females than males. Despite these constraints, with the more accurate and precise effect sizes reported here, researchers can now conduct appropriate power analyses in future work.

Finally, while the present study extended a previous meta-analysis to assess whether bisexual individuals and/or those with intermediate Kinsey scores differ from heterosexual individuals in digit ratios, there is evidence that androgens and estrogens contribute to attraction to males and attraction to females separately (Shirazi et al., 2022; reviewed in Swift-Gallant et al., 2023). Because digit ratios may be influenced by both androgenic and estrogenic signaling (Manning et al., 1998; Zheng and Cohn, 2011), it is possible that combining androphilic and gynephilic attraction in one scale may obscure differences between sexual orientation groups. Thus, future work should consider measuring androphilia and gynephilia separately instead of as ends of a continuum.

### Moderator analyses

Like Grimbos et al., we conducted moderator analyses for geographical location and measurement type. In contrast to Grimbos et al., we did not find consistent effects of geographical location for male digit ratio associations. Specifically, Grimbos et al. found that North American samples had effect sizes in the negative direction, indicating non-heterosexual men have higher digit ratios than heterosexual men, while Europe had effect sizes in the positive direction, indicating non-heterosexual men have lower digit ratios than heterosexual men. In the current analyses, geographical location emerged as a significant moderator, but none of the pairwise comparisons were significant. This may be due to a difference in the number of ethnically diverse samples and/or that these differences may not emerge with the greater number of studies. However, these results do not negate prior work suggesting geographical location/ethnicity differences in digit ratios (e.g., McFadden et al., 2005; Manning and Robinson, 2003), as the majority of these samples are still predominantly White. Geographical location did emerge as a moderator for both the right and left hand heterosexual vs. non-heterosexual women comparison in the present analyses. Left-hand pairwise comparisons indicated that the positive effects were larger for UK/Europe samples compared to North American and others. As Grimbos et al. found that geographical location did not explain more variation than did ethnicity alone, it is likely ethnicity is also driving these effects. As such, it appears critical for future work to consider ethnicity in digit ratio and sexual orientation research (Savolainen et al., 2024).

While measurement type emerged as a moderator for both sexes, pairwise comparisons were significant only for heterosexual vs. non-heterosexual male comparisons. Specifically, these results indicate that larger positive effect sizes (indicating more female-typical digit ratios among non-heterosexual than heterosexual men) were found with the photocopy/scan method than any other method. This may be due to several factors, including that with direct measures the experimenter is likely not completely blinded to the condition and/or could be distracted by their surroundings or the participant in taking the measures. Prior research has also indicated that self-report, compared to photocopy methods, yields smaller effect sizes (Manning et al., 2005; Manning et al., 2007). For these reasons, along with the benefit of including multiple blind raters, it may be advantageous for future research to consider using photocopies/scans when collecting digit ratio data, or expect to increase sample sizes to be adequately powered.

## Conclusion

By examining both published and unpublished datasets, we provide a comprehensive meta-analysis between digit ratios and human sexual orientation in both males and females. These results confirm associations between digit ratios and female sexual orientation, such that same-sex-oriented women tend to have more male-typical ratios than heterosexual women, indicative of higher prenatal androgen exposure among lesbians. In contrast to prior meta-analysis, we also found that both right and left hand digit ratios differed by male sexual orientation, such that homosexual men have more female-typical ratios than heterosexual men. While sexual orientation differences in digit ratios are expected to be smaller than sex differences, we offer several possible limitations to current work that can be addressed in future research. These include the potential existence of subgroups among non-heterosexual individuals that differ in the biological factors contributing to their sexual orientation, as well as the importance of distinguishing between androphilic and gynephilic orientations when investigating the relationship between digit ratios and male sexual orientation.
