# Supplementary material for: Sexual orientation is associated with 2D:4D finger length ratios in both sexes: an updated and expanded meta-analysis
Source: Front Psychol. 2025 Apr 25;16:1559158. doi: 10.3389/fpsyg.2025.1559158 (PMC12062117; doi:10.3389/fpsyg.2025.1559158)
Supplement: Supplementary file 1 [file Table_1.docx]

**Supplementary File**

**
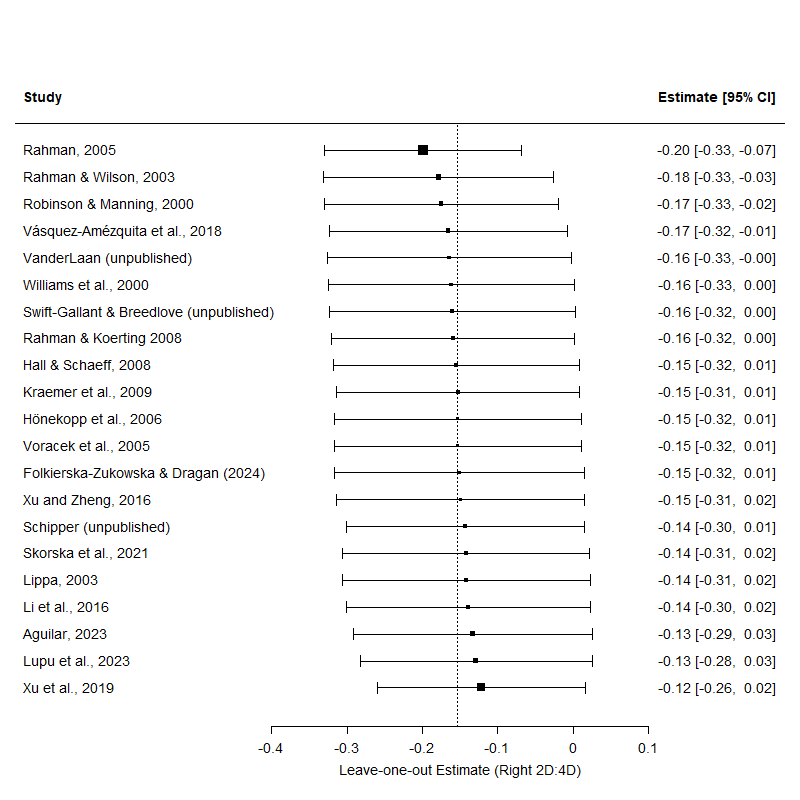
**

**Figure S1.** Leave-one-out sensitivity analysis for right 2D:4D ratios comparison between exclusively heterosexual men and exclusively homosexual men. A vertical dotted line indicates the overall effect.

**
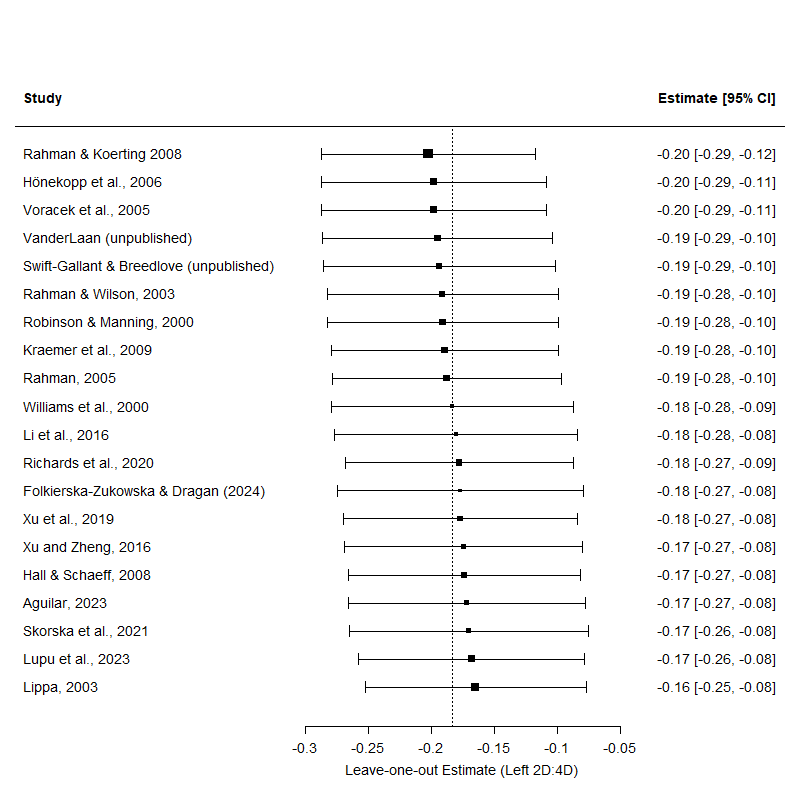
**

**Figure S2.** Leave-one-out sensitivity analysis for left 2D:4D ratios comparison between exclusively heterosexual men and exclusively homosexual men. A vertical dotted line indicates the overall effect.

**
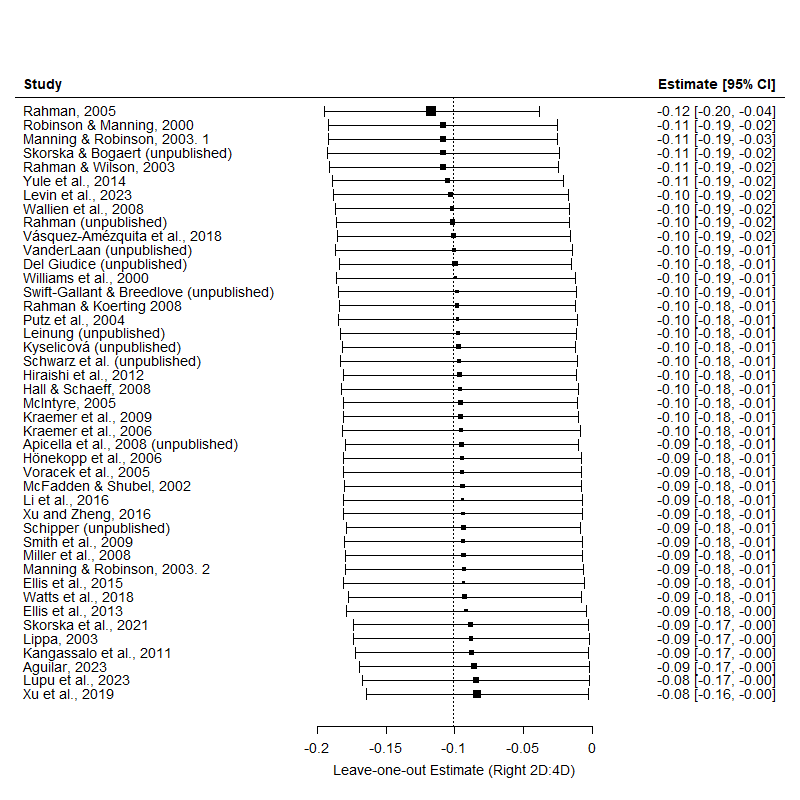
**

**Figure S3.** Leave-one-out sensitivity analysis for right 2D:4D ratios comparison between heterosexual men and non-heterosexual men. A vertical dotted line indicates the overall effect. 1 = UK participants; 2 = Multi-ethnic participants.

**
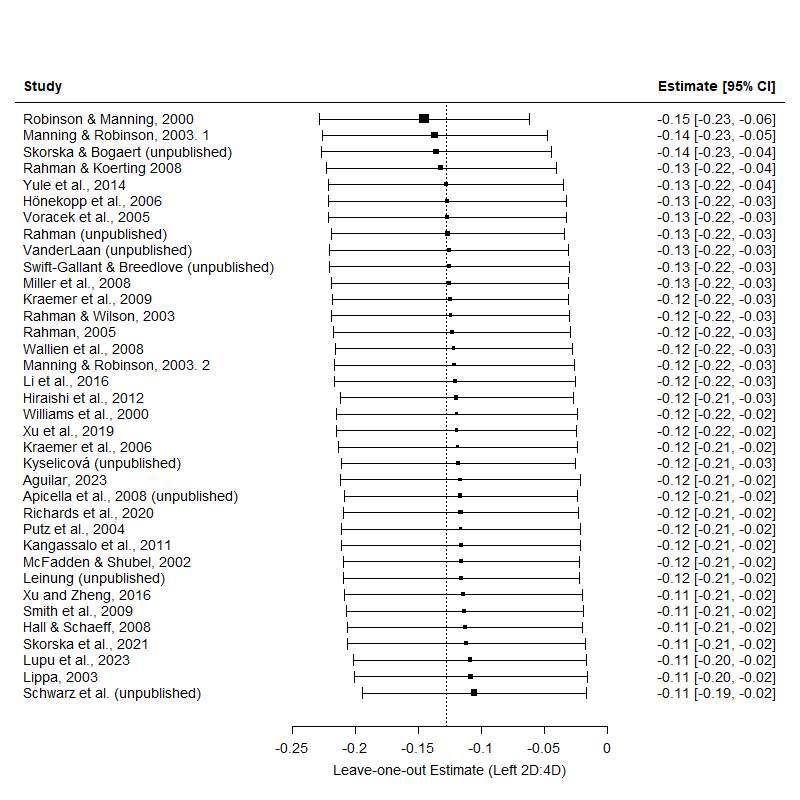
**

**Figure S4.** Leave-one-out sensitivity analysis for left 2D:4D ratios comparison between heterosexual men and non-heterosexual men. A vertical dotted line indicates the overall effect. 1 = UK participants; 2 = Multi-ethnic participants.

**
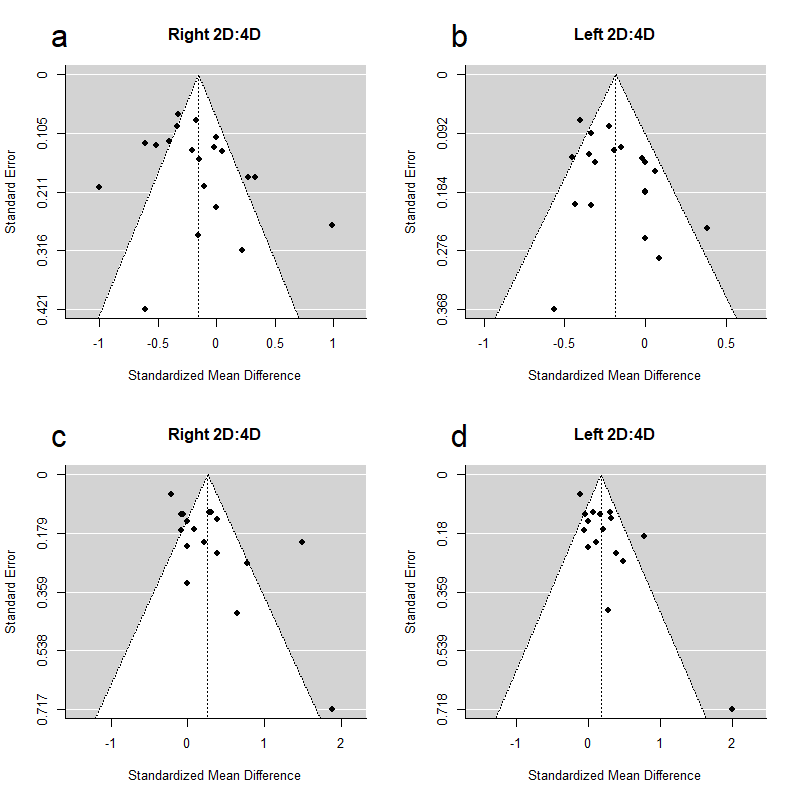
**

**Figure S5.** Funnel plot for studies examining (a) right 2D:4D ratios difference in exclusively heterosexual men and homosexual men, (b) left 2D:4D ratios difference in exclusively heterosexual men and homosexual men, (c) right 2D:4D ratios difference in exclusively heterosexual women and homosexual women, and (d) left 2D:4D ratios difference in exclusively heterosexual women and homosexual women.

**
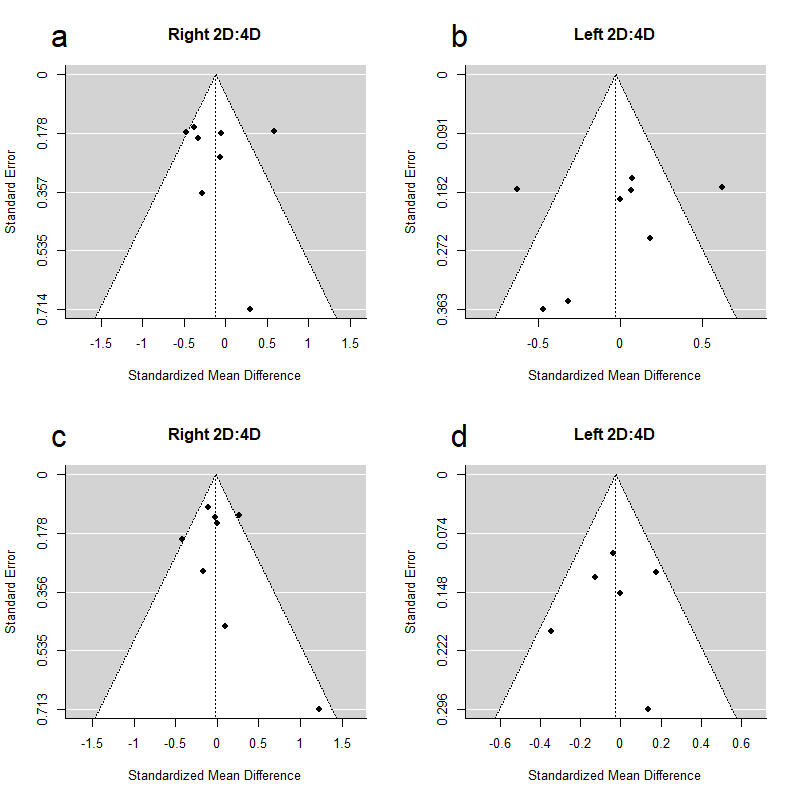
**

**Figure S6.** Funnel plot for studies examining (a) right 2D:4D ratios difference in heterosexual men and bisexual men, (b) left 2D:4D ratios difference in heterosexual men and bisexual men, (c) right 2D:4D ratios difference in heterosexual women and bisexual women, and (d) left 2D:4D ratios difference in heterosexual women and bisexual women.

**
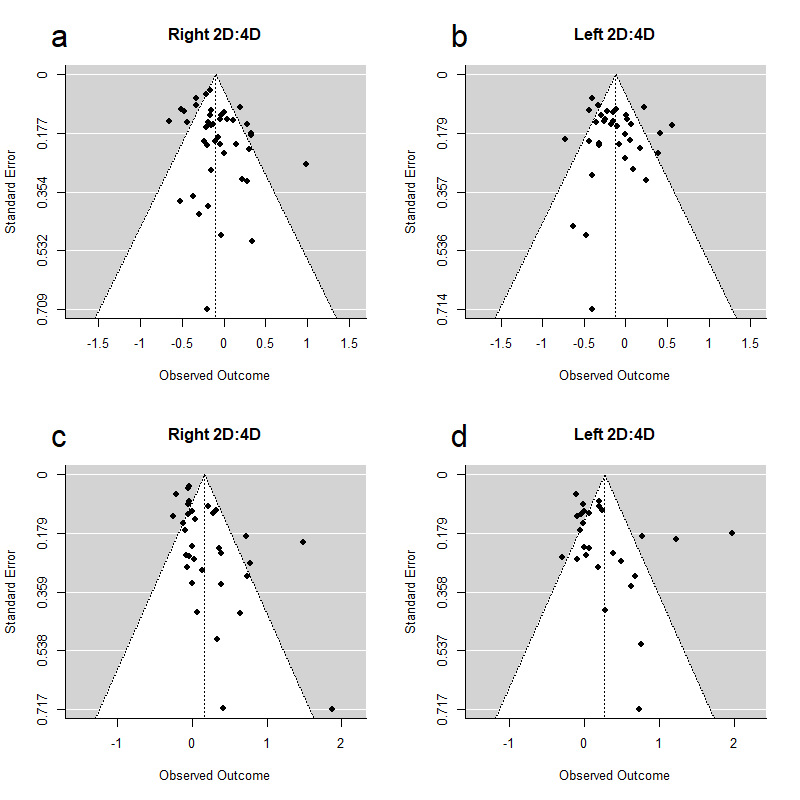
**

**Figure S7.** Funnel plot for studies examining (a) right 2D:4D ratios difference in heterosexual men and non-heterosexual men, (b) left 2D:4D ratios difference in heterosexual men and non-heterosexual men, (c) right 2D:4D ratios difference in heterosexual women and non-heterosexual women, and (d) left 2D:4D ratios difference in heterosexual women and non-heterosexual women.

**
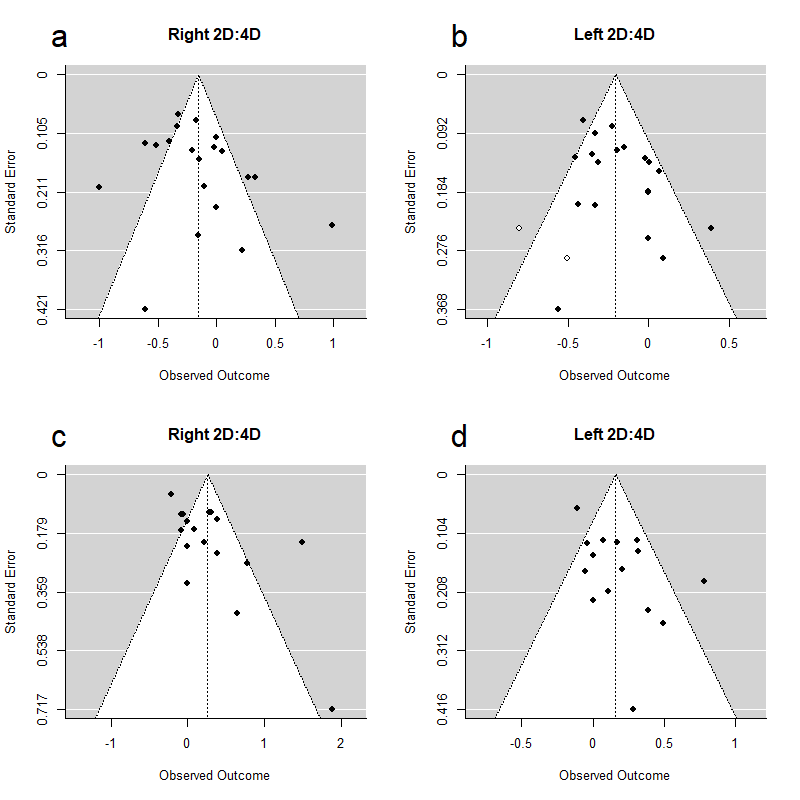
**

**Figure S8.** Funnel plot with Duval’s trim-and-fill correction for studies examining (a) right 2D:4D ratios difference in exclusively heterosexual men and homosexual men (*k* = 0 imputed study; (*g =* -0.15 [-0.31, 0.00], *p* = 0.052)), (b) left 2D:4D ratios difference in exclusively heterosexual men and homosexual men (*k* = 2 imputed studies; *g =* -0.20 [-0.30, -0.11], *p* < 0.001), (c) right 2D:4D ratios difference in exclusively heterosexual women and homosexual women (*k* = 0 imputed study; *g =* 0.26 [0.05, 0.47], *p* = 0.016), and (d) left 2D:4D ratios difference in exclusively heterosexual women and homosexual women (*k* = 0 imputed study; *g =* 0.16 [0.04, 0.28], *p* = 0.010). Unfilled circles indicate imputed missing studies, produced by the L0 estimator and Egger’s regression test.

**
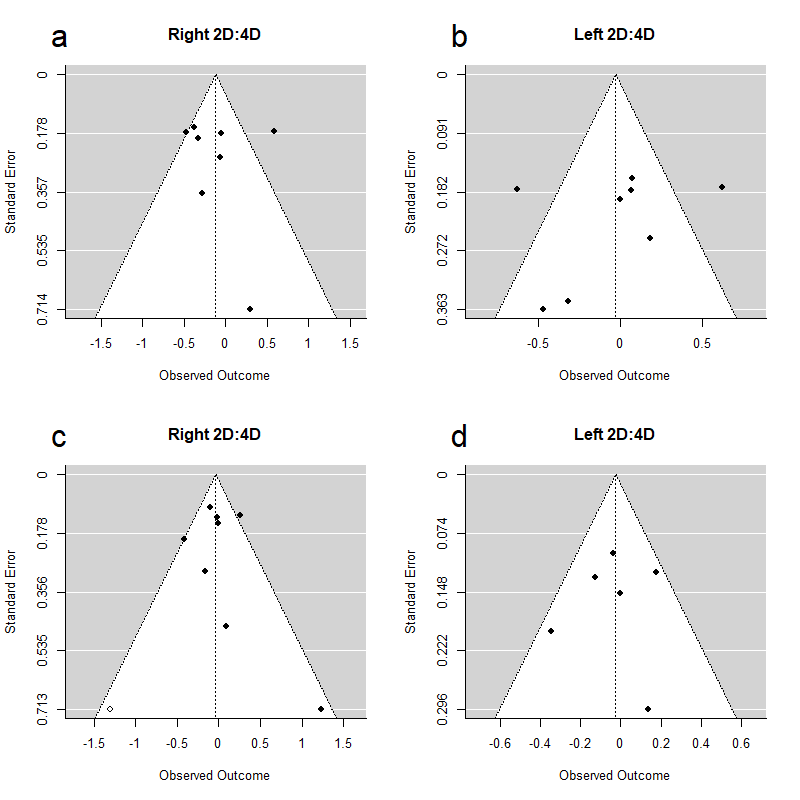
**

**Figure S9.** Funnel plot with Duval’s trim-and-fill correction for studies examining (a) right 2D:4D ratios difference in heterosexual men and bisexual men (*k* = 0 imputed study; *g =* -0.12 [-0.40, 0.16], *p* = 0.401), (b) left 2D:4D ratios difference in heterosexual men and bisexual men (*k* = 0 imputed study; *g =* -0.03 [-0.32, 0.26], *p* = 0.850), (c) right 2D:4D ratios difference in heterosexual women and bisexual women (*k* = 1 imputed study; *g =* -0.04 [-0.21, 0.13], *p* = 0.681), and (d) left 2D:4D ratios difference in heterosexual women and bisexual women (*k* = 0 imputed study; *g =* -0.02 [-0.15, 0.10], *p* = 0.703). Unfilled circles indicate imputed missing studies, produced by the L0 estimator and Egger’s regression test. Standard errors were not initially estimated for comparisons in men, and the side of the funnel plot was specified.

**
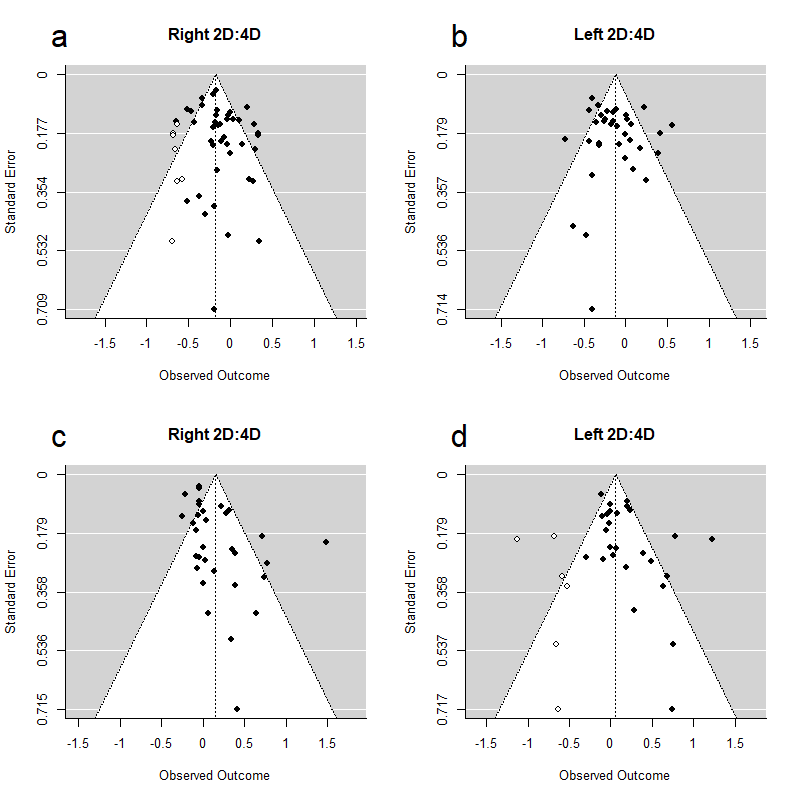
**

**Figure S10.** Funnel plot with Duval’s trim-and-fill correction for studies examining (a) right 2D:4D ratios difference in heterosexual men and non-heterosexual men (*k* = 7 imputed studies; *g =* -0.17 [-0.26, -0.09], *p* < 0.001), (b) left 2D:4D ratios difference in heterosexual men and non-heterosexual men (*k* = 0 imputed study; *g =* -0.12 [-0.21, -0.03], *p* = 0.010), (c) right 2D:4D ratios difference in heterosexual women and non-heterosexual women (*k* = 0 imputed study; *g =* 0.15 [0.03, 0.28], *p* = 0.019), and (d) left 2D:4D ratios difference in heterosexual women and non-heterosexual women (*k* = 6 imputed studies; *g =* 0.06 [-0.10, 0.22], *p* = 0.472). Unfilled circles indicate imputed missing studies, produced by the L0 estimator and Egger’s regression test

**
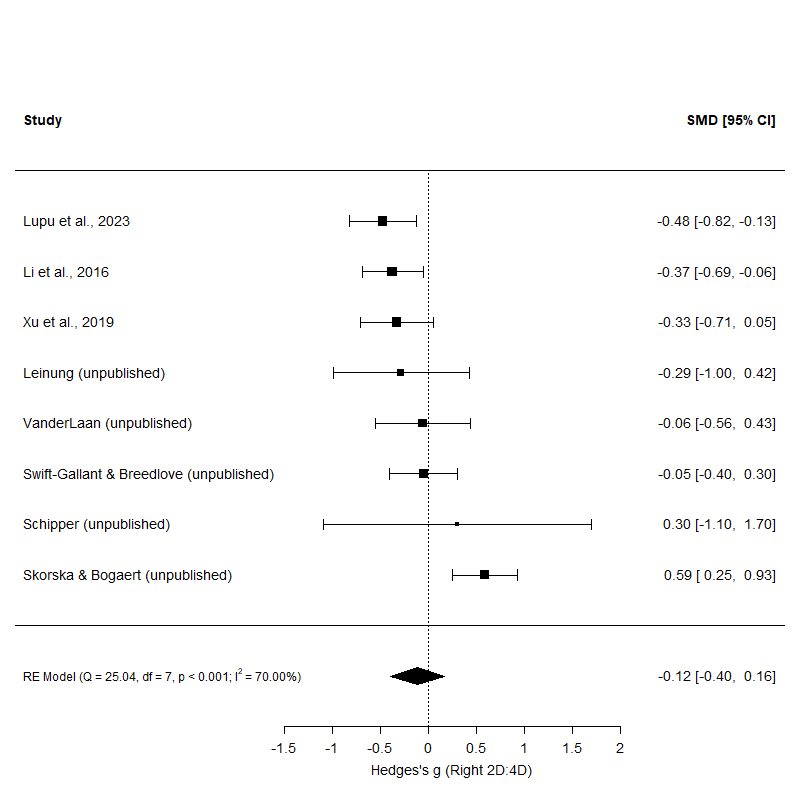
**

**Figure S11.** Right 2D:4D ratios comparison between heterosexual men and bisexual men

**
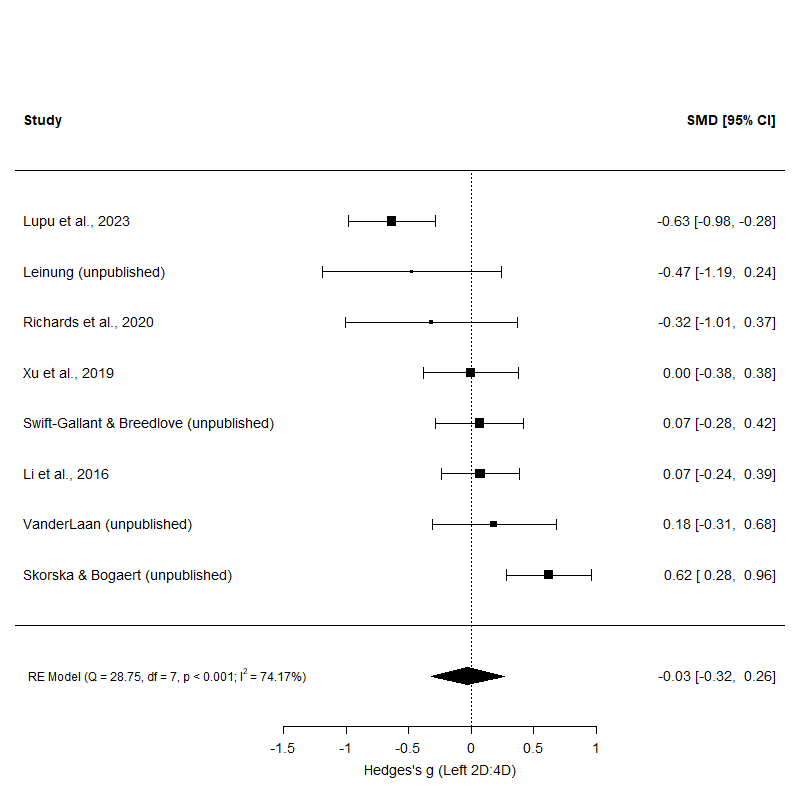
**

**Figure S12.** Left 2D:4D ratios comparison between heterosexual men and bisexual men

**
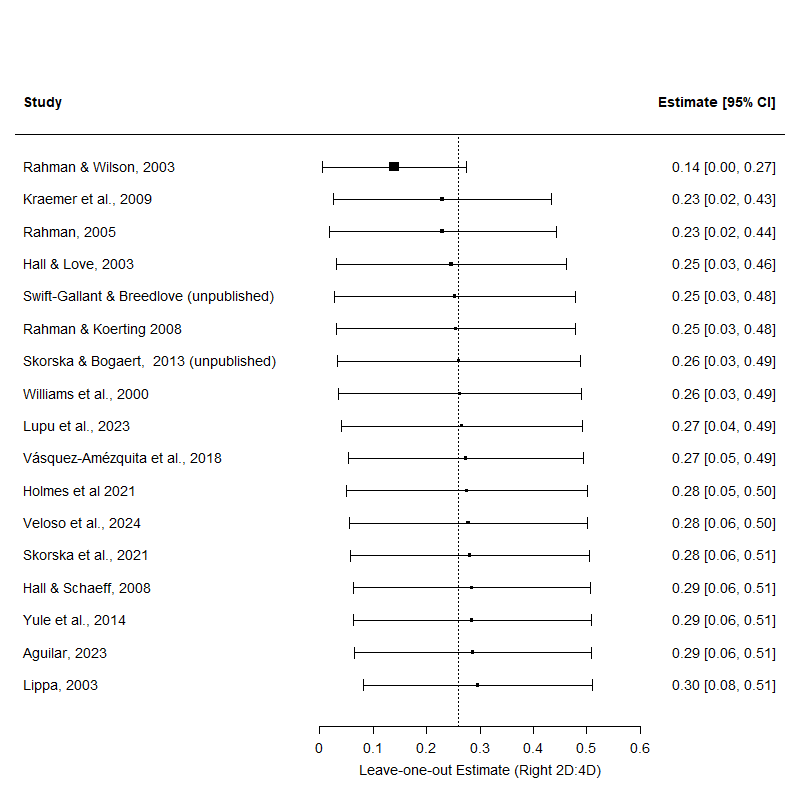
**

**Figure S13.** Leave-one-out sensitivity analysis for right 2D:4D ratios comparison between exclusively heterosexual women and exclusively homosexual women. A vertical dotted line indicates the overall effect.

**
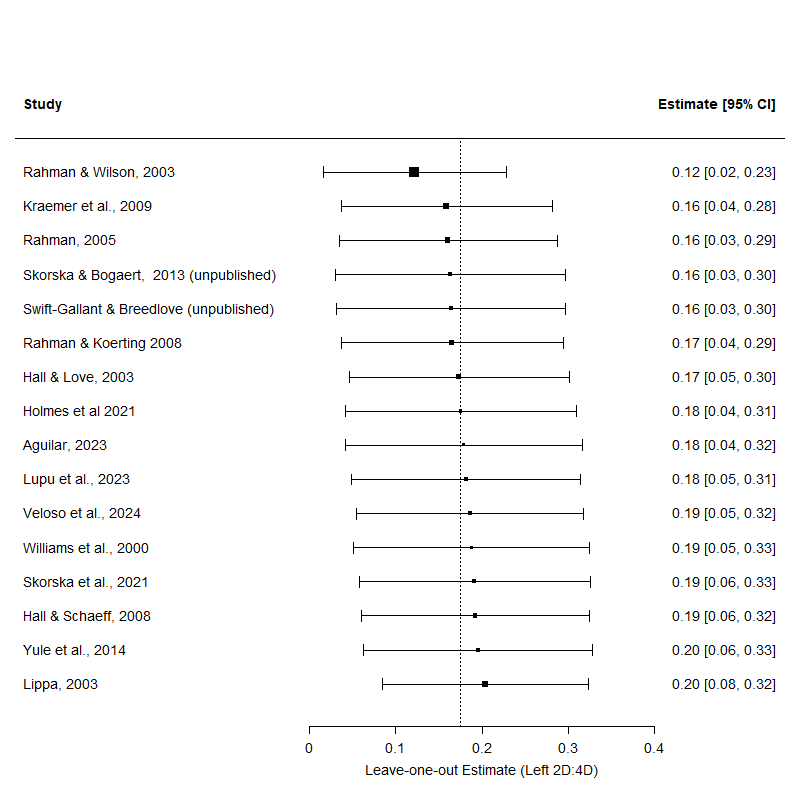
**

**Figure S14.** Leave-one-out sensitivity analysis for left 2D:4D ratios comparison between exclusively heterosexual women and exclusively homosexual women. A vertical dotted line indicates the overall effect.

**
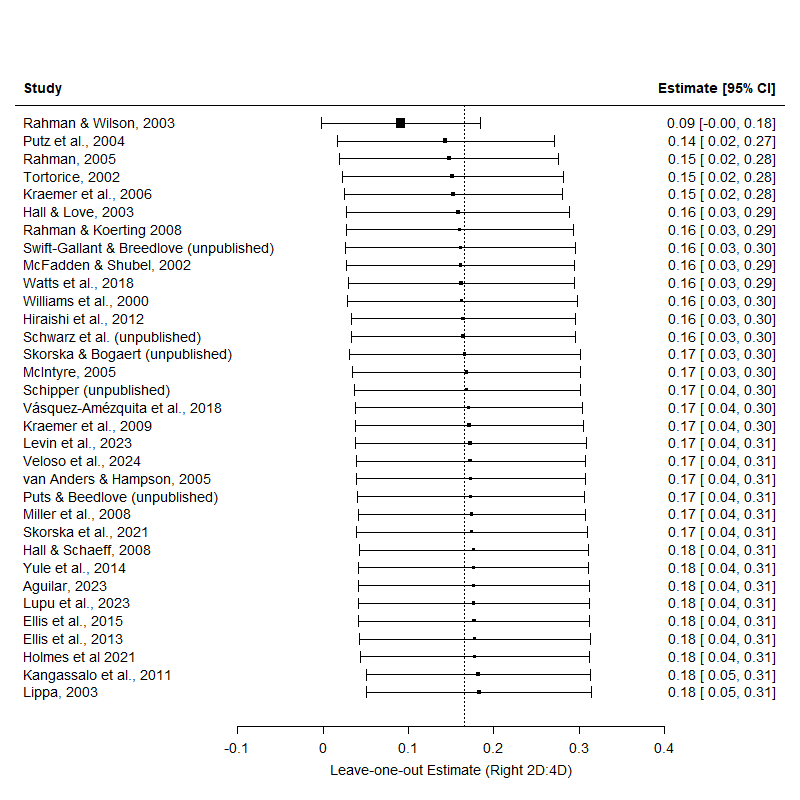
**

**Figure S15.** Leave-one-out sensitivity analysis for right 2D:4D ratios comparison between heterosexual women and non-heterosexual women. A vertical dotted line indicates the overall effect.

**
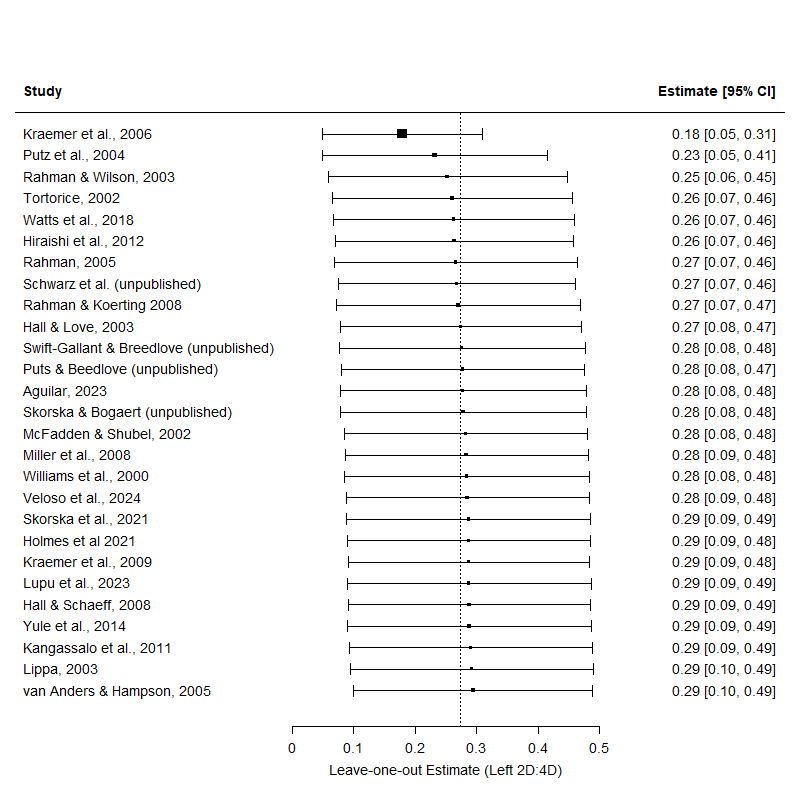
**

**Figure S16.** Leave-one-out sensitivity analysis for left 2D:4D ratios comparison between heterosexual women and non-heterosexual women. A vertical dotted line indicates the overall effect.

**
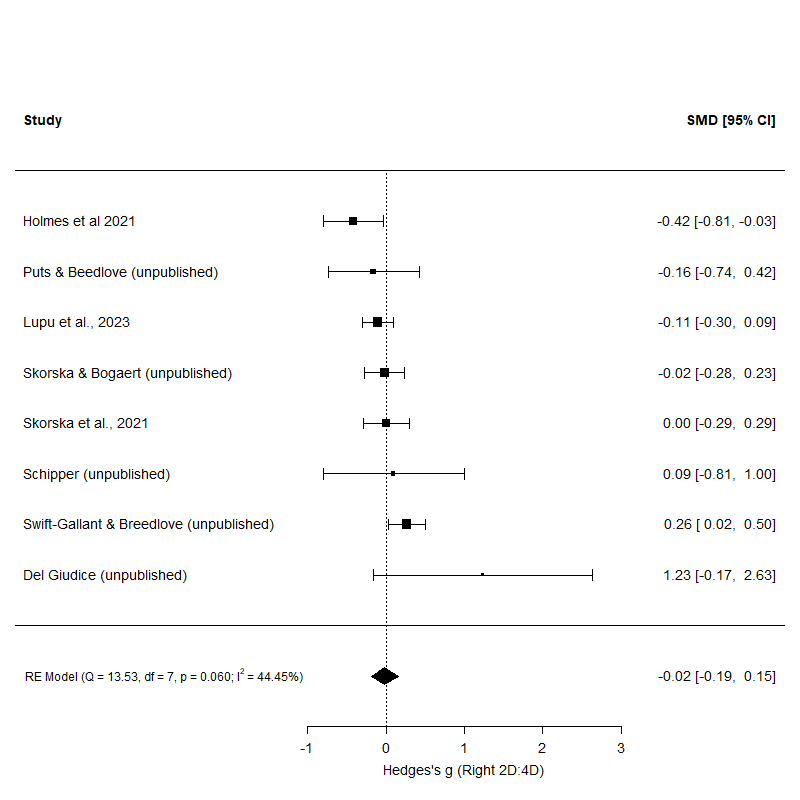
**

**Figure S17.** Right 2D:4D ratios comparison between heterosexual women and bisexual women


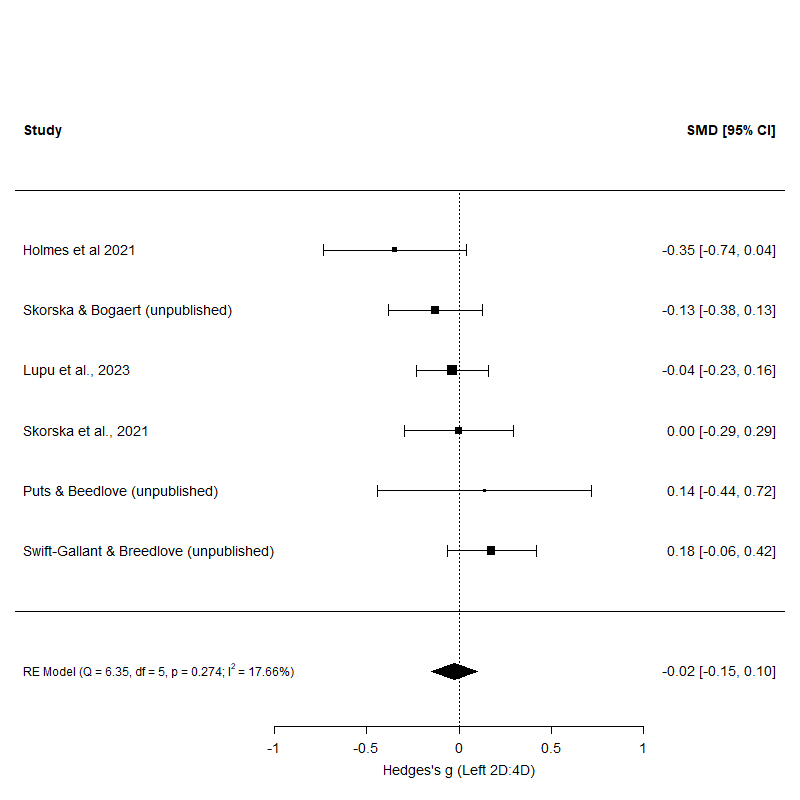


**Figure S18.** Left 2D:4D ratios comparison between heterosexual women and bisexual women

**
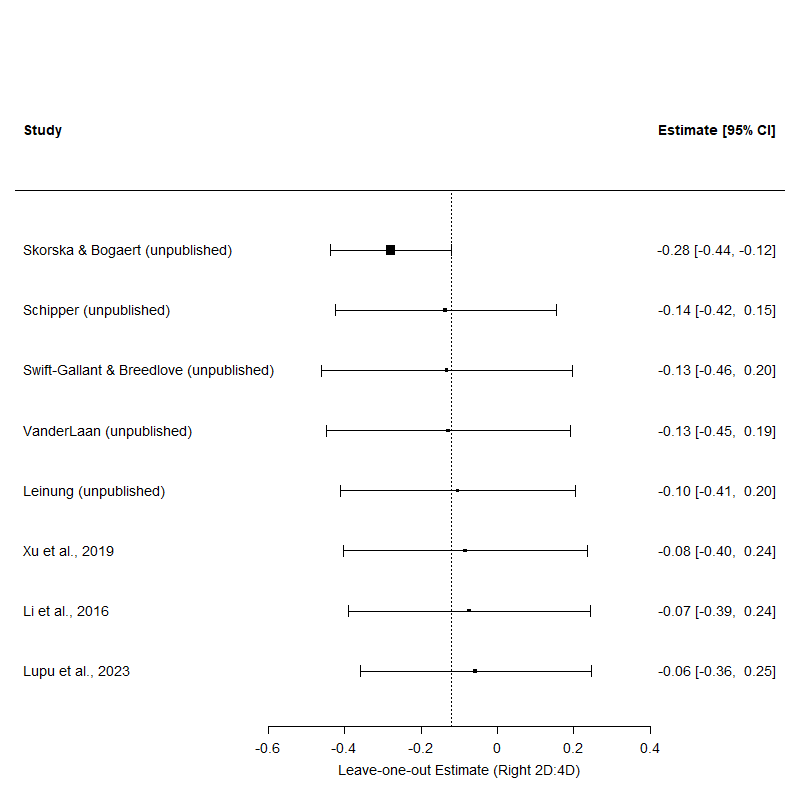
**

**Figure S19.** Leave-one-out sensitivity analysis for right 2D:4D ratios comparison between exclusively heterosexual men and bisexual men. A vertical dotted line indicates the overall effect.

**
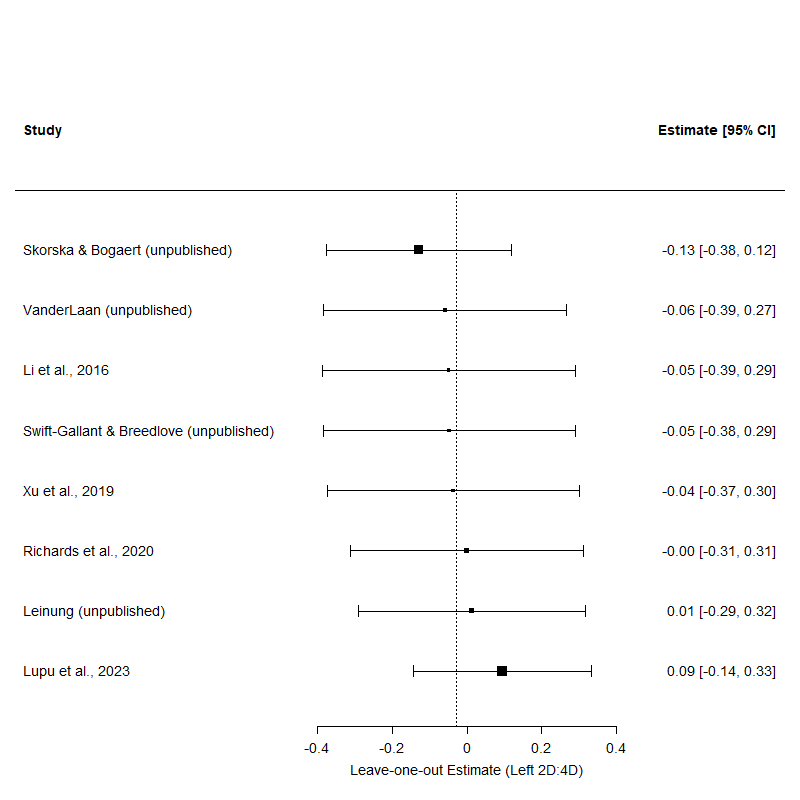
**

**Figure S20.** Leave-one-out sensitivity analysis for left 2D:4D ratios comparison between exclusively heterosexual men and bisexual men. A vertical dotted line indicates the overall effect.

**
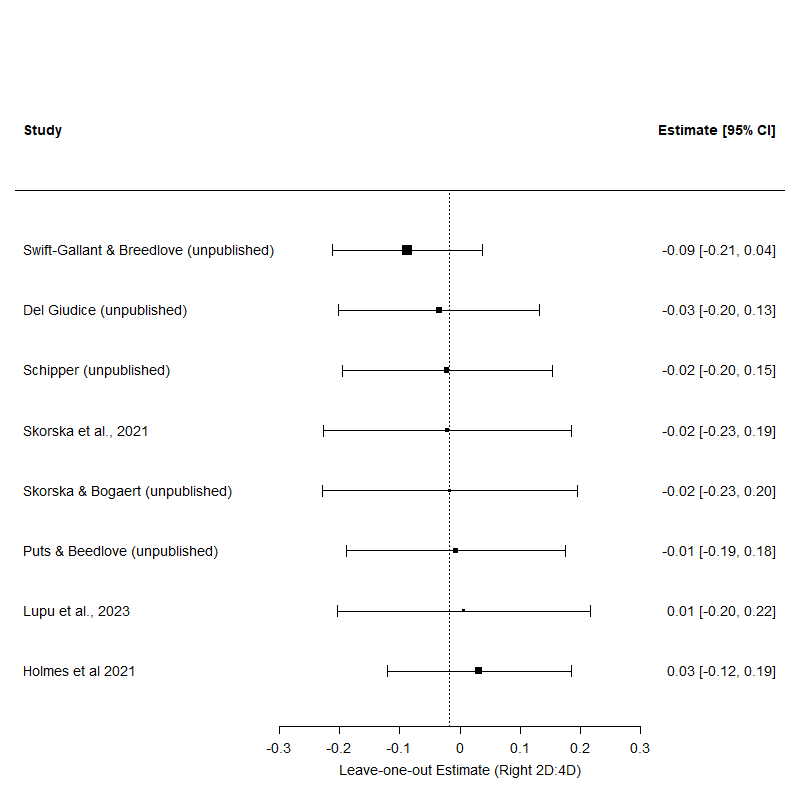
**

**Figure S21.** Leave-one-out sensitivity analysis for right 2D:4D ratios comparison between exclusively heterosexual women and bisexual women. A vertical dotted line indicates the overall effect.

**
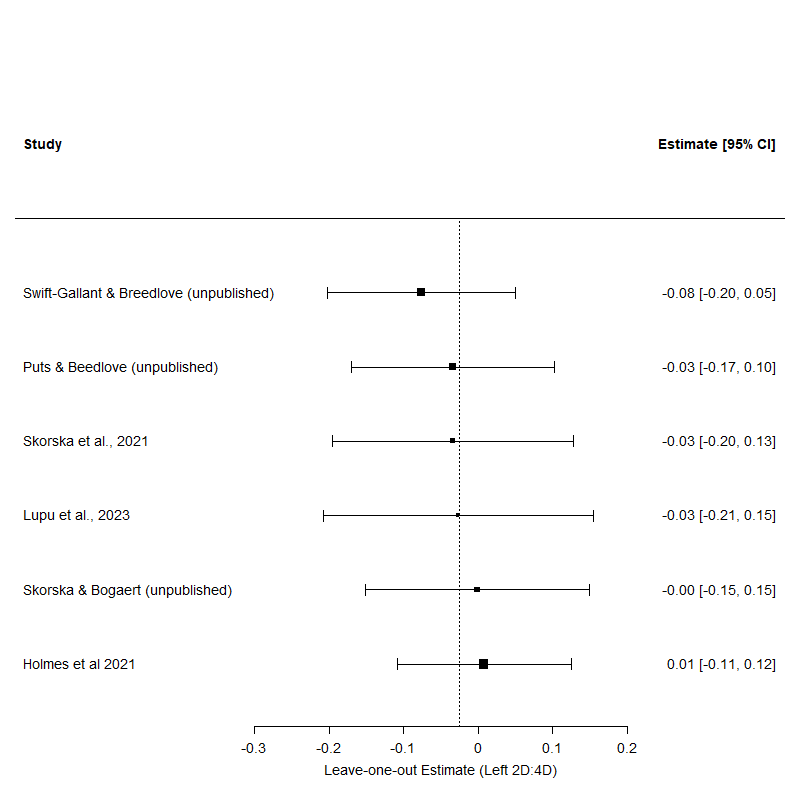
Figure S22.** Leave-one-out sensitivity analysis for left 2D:4D ratios comparison between exclusively heterosexual women and bisexual women. A vertical dotted line indicates the overall effect.

Table S1: Excluded studies

| **Exclusion criteria** | **Studies excluded** |
| --- | --- |
| 1. Studies that did not provide digit ratio data by sex and/or sexual orientation | - Veale et al., 2010 - Turan et al., 2021 - Richards et al., 2020 - Manning et al., 2005 - Hall & Lujan (unpublished) |
| 1. focused exclusively on heterosexual or homosexual individuals | - Holmes et al., 2022a - Rahman et al., 2005 - Fussell et al., 2011 - Manning et al., 2023 - McIntyre et al., 2003 - Swift-Gallant et al., 2021 - Vinhal Nepomuceno et al., 2015 (unpublished) |
| 1. used or reanalyzed previously published data that was already selected for inclusion in meta-analysis | - Ellis et al., 2017 - Collaer et al., 2007 - Holmes et al., 2022b - Manning et al., 2017 - Manning et al., 2022 - Martin et al., 2008 - McFadden & Shubel, 2003 - McFadden et al., 2005 - Peters et al., 2007 - Rahman et al., 2004 |

**Table S2.** Results from moderator analyses for heterosexual men vs. bisexual men

| **Right 2D:4D Model** | **Q_m_** | **k** | **g** | **se** | ***z*** | ***p*** | **Lower CI** | **Upper CI** |
| --- | --- | --- | --- | --- | --- | --- | --- | --- |
| **Geographic Location** | 5.74 |  |  |  |  | 0.125 |  |  |
| North America |  | 5 | 0.12 | 0.16 | 0.74 | 0.461 | -0.20 | 0.44 |
| UK/Europe |  | 2 | -0.41 | 0.22 | -1.88 | 0.060 | -0.83 | 0.02 |
| Other^+^ |  |  |  |  |  |  |  |  |
| Asia |  | 1 | -0.37 | 0.29 | -1.28 | 0.199 | -0.95 | 0.20 |
| **Measurement Type**^1^ | 9.55 |  |  |  |  | **0.023** |  |  |
| Direct |  | 2 | 0.33 | 0.19 | 1.76 | 0.078 | -0.04 | 0.71 |
| Self-report^+^ |  |  |  |  |  |  |  |  |
| Photocopy/scan |  | 5 | -0.29 | 0.12 | -2.44 | **0.015** | -0.53 | -0.06 |
| Mixed or unknown |  | 1 | -0.29 | 0.40 | -0.72 | 0.472 | -1.07 | 0.50 |
| **Publication status**^2^ | 7.59 |  |  |  |  | 0.023 |  |  |
| Published |  | 3 | -0.40 | 0.15 | -2.61 | **0.009** | -0.69 | -0.10 |
| Unpublished |  | 5 | 0.13 | 0.15 | 0.89 | 0.372 | -0.16 | 0.42 |
|  |  |  |  |  |  |  |  |  |
| **Left 2D:4D Model** |  | k | **g** | **se** | ***z*** | ***p*** | **Lower CI** | **Upper CI** |
| **Geographic Location** | 1.49 |  |  |  |  | 0.685 |  |  |
| North America |  | 5 | 0.09 | 0.20 | 0.43 | 0.664 | -0.30 | 0.47 |
| UK/Europe |  | 2 | -0.32 | 0.29 | -1.12 | 0.261 | -0.88 | 0.24 |
| Other^+^ |  |  |  |  |  |  |  |  |
| Asia |  | 1 | 0.07 | 0.39 | 0.19 | 0.852 | -0.70 | 0.84 |
| **Measurement Type** | 5.14 |  |  |  |  | 0.273 |  |  |
| Direct |  | 2 | 0.43 | 0.25 | 1.74 | 0.082 | -0.05 | 0.92 |
| Self-report |  | 1 | -0.32 | 0.45 | -0.71 | 0.476 | -1.20 | 0.56 |
| Photocopy/scan |  | 4 | -0.12 | 0.17 | -0.73 | 0.464 | -0.45 | 0.20 |
| Mixed or unknown |  | 1 | -0.47 | 0.46 | -1.03 | 0.301 | -1.37 | 0.42 |
| **Publication status** | 1.96 |  |  |  |  | 0.375 |  |  |
| Published |  | 4 | -0.21 | 0.19 | -1.09 | 0.275 | -0.58 | 0.16 |
| Unpublished |  | 4 | 0.17 | 0.20 | 0.88 | 0.381 | -0.21 | 0.55 |

**Note.** Pairwise comparisons were tested for the model with a significant moderator. ^1^Significant difference was observed between photocopy/scan and direct measures (*p* = .005). ^2^Significant difference was observed between published and unpublished studies (*p* = .013). ^+^Not Identified in data.

**Table S3.** Results from moderator analyses for heterosexual women vs. bisexual women

| **Right 2D:4D Model** | **Q_m_** | **k** | **g** | **se** | ***z*** | ***p*** | **Lower CI** | **Upper CI** |
| --- | --- | --- | --- | --- | --- | --- | --- | --- |
| **Geographic Location** | 1.88 |  |  |  |  | 0.598 |  |  |
| North America |  | 4 | 0.08 | 0.12 | 0.71 | 0.475 | -0.15 | 0.32 |
| UK/Europe |  | 3 | -0.16 | 0.14 | -1.17 | 0.242 | -0.43 | 0.11 |
| Other^+^ |  |  |  |  |  |  |  |  |
| Asia |  | 1 | 0.00 | 0.20 | 0.00 | 1.000 | -0.40 | 0.40 |
| **Measurement Type** | 0.03 |  |  |  |  | 0.983 |  |  |
| Direct |  | 2 | -0.01 | 0.17 | -0.07 | 0.943 | -0.34 | 0.31 |
| Self-report^+^ |  |  |  |  |  |  |  |  |
| Photocopy/scan |  | 6 | -0.02 | 0.12 | -0.17 | 0.864 | -0.26 | 0.21 |
| Mixed or unknown^+^ |  |  |  |  |  |  |  |  |
| **Publication status** | 3.06 |  |  |  |  | 0.217 |  |  |
| Published |  | 3 | -0.14 | 0.10 | -1.36 | 0.174 | -0.34 | 0.06 |
| Unpublished |  | 5 | 0.12 | 0.10 | 1.10 | 0.272 | -0.09 | 0.32 |
|  |  |  |  |  |  |  |  |  |
| **Left 2D:4D Model** |  | **k** | **g** | **se** | ***z*** | ***p*** | **Lower CI** | **Upper CI** |
| **Geographic Location** | 1.13 |  |  |  |  | 0.770 |  |  |
| North America |  | 3 | 0.04 | 0.12 | 0.36 | 0.721 | -0.20 | 0.29 |
| UK/Europe |  | 2 | -0.14 | 0.14 | -1.00 | 0.317 | -0.41 | 0.13 |
| Other^+^ |  |  |  |  |  |  |  |  |
| Asia |  | 1 | 0.00 | 0.20 | 0.00 | 1.000 | -0.40 | 0.40 |
| **Measurement Type** | 0.34 |  |  |  |  | 0.845 |  |  |
| Direct |  | 2 | -0.07 | 0.12 | -0.58 | 0.562 | -0.31 | 0.17 |
| Self-report^+^ |  |  |  |  |  |  |  |  |
| Photocopy/scan |  | 4 | 0.00 | 0.09 | -0.04 | 0.972 | -0.18 | 0.17 |
| Mixed or unknown^+^ |  |  |  |  |  |  |  |  |
| **Publication status** | 0.95 |  |  |  |  | 0.621 |  |  |
| Published |  | 3 | -0.09 | 0.10 | -0.89 | 0.375 | -0.28 | 0.10 |
| Unpublished |  | 3 | 0.04 | 0.11 | 0.40 | 0.687 | -0.16 | 0.25 |

**Note.** ^+^Not identified in data.

**Table S4.** Results from moderator analyses for heterosexual, bisexual, and homosexual men

| **Right 2D:4D Model** | **Q_m_** | **k** | **g** | **se** | ***z*** | ***p*** | **Lower CI** | **Upper CI** |
| --- | --- | --- | --- | --- | --- | --- | --- | --- |
| **Comparison Type** | 7.19 |  |  |  |  | 0.066 |  |  |
| Bisexual vs. Homosexual |  | 5 | 0.11 | 0.12 | 0.89 | 0.374 | -0.13 | 0.35 |
| Heterosexual vs. Bisexual |  | 8 | -0.02 | 0.11 | -0.20 | 0.838 | -0.24 | 0.19 |
| Heterosexual vs. Homosexual |  | 21 | -0.13 | 0.08 | -1.74 | 0.082 | -0.28 | 0.02 |
|  |  |  |  |  |  |  |  |  |
| **Left 2D:4D Model** |  | **k** | **g** | **se** | ***z*** | ***p*** | **Lower CI** | **Upper CI** |
| **Comparison Type**^1^ | 11.22 |  |  |  |  | **0.011** |  |  |
| Bisexual vs. Homosexual |  | 6 | -0.13 | 0.10 | -1.29 | 0.196 | -0.34 | 0.07 |
| Heterosexual vs. Bisexual |  | 8 | -0.01 | 0.09 | -0.07 | 0.941 | -0.18 | 0.17 |
| Heterosexual vs. Homosexual |  | 20 | -0.17 | 0.05 | -3.18 | **0.002** | -0.27 | -0.06 |

**Note.** ^1^No significant difference was observed for all pairwise other comparisons (*p* > .05).

**Table S5.** Results from moderator analyses for heterosexual, bisexual, and homosexual women

| **Right 2D:4D Model** | **Q_m_** | **k** | **g** | **se** | ***z*** | ***p*** | **Lower CI** | **Upper CI** |
| --- | --- | --- | --- | --- | --- | --- | --- | --- |
| **Comparison Type**^1^ | 12.27 |  |  |  |  | **0.007** |  |  |
| Bisexual vs. Homosexual |  | 5 | 0.29 | 0.13 | 2.31 | **0.021** | 0.04 | 0.54 |
| Heterosexual vs. Bisexual |  | 8 | 0.01 | 0.11 | 0.14 | 0.893 | -0.20 | 0.23 |
| Heterosexual vs. Homosexual |  | 17 | 0.24 | 0.09 | 2.64 | **0.008** | 0.06 | 0.42 |
|  |  |  |  |  |  |  |  |  |
| **Left 2D:4D Model** |  |  |  |  |  |  |  |  |
| **Comparison Type**^1^ | 11.43 |  |  |  |  | **0.010** |  |  |
| Bisexual vs. Homosexual |  | 5 | 0.19 | 0.09 | 2.03 | **0.042** | 0.01 | 0.37 |
| Heterosexual vs. Bisexual |  | 6 | -0.03 | 0.08 | -0.37 | 0.712 | -0.19 | 0.13 |
| Heterosexual vs. Homosexual |  | 16 | 0.16 | 0.06 | 2.72 | **0.007** | 0.04 | 0.27 |

**Note.** ^1^Significant difference was observed between Bisexual vs. Homosexual and Heterosexual vs. Bisexual, and between Heterosexual vs. Bisexual and Heterosexual vs. Homosexual (*p* < .05).

**Supplementary Results**

The following results include the same primary analyses as included in the main manuscript but with Manning et al., 2007 included. Overall, the findings align with the results presented in the main manuscript.

**Sex Differences in Digit Ratios**

Digit ratios exhibited expected sex differences: Heterosexual men had lower 2D:4D than heterosexual women for both the right hand (*g =* -0.45, *p* < 0.001; Fig. 2) and left hand (*g =* -0.41, *p* < 0.001).

**Male Sexual Orientation and Digit Ratios**

***Exclusive heterosexual vs. exclusive homosexual men***

Exclusively heterosexual men had a lower right (*g =* -0.15, *p* = 0.045) and left hand 2D:4D (*g =* -0.17, *p* < 0.001) than exclusively homosexual men.

***Heterosexual vs. nonheterosexual men***

Exclusively heterosexual men had a lower right 2D:4D (*g =* -0.10, *p* = 0.015) and a lower left hand 2D:4D (*g =* -0.13, *p* = 0.005) than nonheterosexual (bisexual plus homosexual) men.

***Heterosexual vs. bisexual men***

Exclusively heterosexual men did not differ from bisexual men for right (*g =* -0.11, *p* = 0.371) or left (*g =* -0.04, *p* = 0.751) 2D:4D.

**Female Sexual Orientation and Digit Ratios**

***Exclusive heterosexual vs. exclusive homosexual women***

Exclusively heterosexual women had a higher right 2D:4D (*g =* 0.24, *p* = 0.016) and a higher left 2D:4D (*g =* 0.16, *p* = 0.007) than exclusively homosexual women.

***Heterosexual vs. nonheterosexual women***

Heterosexual women had a higher digit ratio than nonheterosexual women in both right (*g =* 0.16, *p* = 0.010) and left (*g =* 0.26, *p* = 0.005) hands.

***Heterosexual vs. bisexual women***

Neither right 2D:4D (*g =* -0.01, *p* = 0.866) nor left 2D:4D (*g =* -0.001, *p* = 0.931) differed between exclusively heterosexual women and bisexual women.
